# Eculizumab for adult patients with atypical haemolytic-uraemic syndrome: full dataset analysis of Japanese post-marketing surveillance

**DOI:** 10.1007/s40620-024-01921-y

**Published:** 2024-05-29

**Authors:** Shoichi Maruyama, Yoichiro Ikeda, Shinya Kaname, Noritoshi Kato, Masanori Matsumoto, Yumiko Ishikawa, Akihiko Shimono, Yoshitaka Miyakawa, Masaomi Nangaku, Yugo Shibagaki, Hirokazu Okada

**Affiliations:** 1 Department of Nephrology, Nagoya University Graduate School of Medicine, 65 Tsurumai-cho, Showa-ku, Nagoya, Aichi 466-8550 Japan; 2 Division of Nephrology and Endocrinology, The University of Tokyo, 7-3-1, Hongo, Bunkyo-ku, Tokyo, 113-8655 Japan; 3 Department of Nephrology and Rheumatology, Kyorin University School of Medicine, 6-20-2 Shinkawa, Mitaka-City, Tokyo 181-8611 Japan; 4 Department of Blood Transfusion Medicine, Nara Medical University, 840 Shijyo-cho, Kashihara City, Nara 634-8522 Japan; 5 Alexion Pharma GK, 3-1-1 Shibaura, Minato-Ku, Tokyo, 108-0023 Japan; 6 Department of Haematology, Saitama Medical University, 38 Moroyama, Iruma-gun, Saitama, 350-0495 Japan; 7 Division of Nephrology and Hypertension, Department of Medicine, St. Marianna University School of Medicine, 2-16-1 Sugao, Miyamae-ku, Kawasaki, Kanagawa 216-8511 Japan; 8 Department of Nephrology, Saitama Medical University, 38 Moroyama, Iruma-Gun, Saitama, 350-0495 Japan

**Keywords:** Atypical haemolytic uraemic syndrome, Thrombotic microangiopathy, Eculizumab, Post-marketing surveillance

## Abstract

**Background:**

Eculizumab has been approved for atypical haemolytic-uraemic syndrome (aHUS) in Japan since 2013. Post-marketing surveillance enrolled patients with aHUS who received ≥ 1 dose of eculizumab to assess eculizumab safety and effectiveness.

**Methods:**

We evaluated serious adverse events and effectiveness endpoints, i.e., haematologic normalization, a decrease of ≥ 25% in serum creatinine (sCr) levels, and complete thrombotic microangiopathy (TMA) response in adult patients with aHUS without other underlying diseases. In addition, the difference of baseline characteristics between patients who did and did not meet effectiveness endpoints was examined.

**Results:**

In this safety and effectiveness analysis, 79 adult patients were included; median age was 54.0 years, median treatment duration was 30 weeks. Total exposure time of eculizumab was 75.51 patient-years, and 94 serious adverse events were reported in 39 patients. No unexpected safety signals were identified in this population. Mean platelet count, lactate dehydrogenase and estimated glomerular filtration rate significantly improved after 7 days of treatment. Complete TMA response, haematologic normalization and the improvement of sCr levels were met by 35.3%, 40.4% and 51.3% of patients, respectively. Median treatment duration was shorter in patients who did not achieve complete TMA response (6 weeks) than in patients who did (114 weeks). Multivariate analysis suggested that the time from the most recent TMA episode to start of eculizumab treatment was negatively associated with kidney function improvement.

**Conclusions:**

No unexpected safety signals of eculizumab were identified in Japanese patients with aHUS in a real-world setting. Renal outcomes were negatively associated with the time from the most recent TMA episode to the initiation of eculizumab treatment.

**Graphical abstract:**

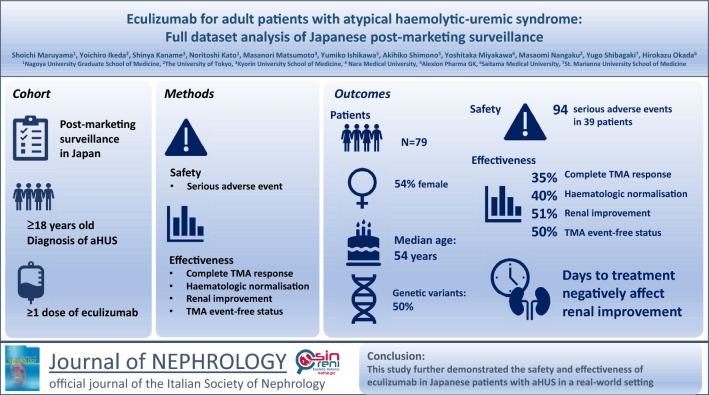

**Supplementary Information:**

The online version contains supplementary material available at 10.1007/s40620-024-01921-y.

## Introduction

Atypical haemolytic-uraemic syndrome (aHUS) is a form of thrombotic microangiopathy (TMA) associated with dysregulation of the alternative complement pathway [1–3]. It is characterized by microangiopathic haemolytic anaemia (MAHA), thrombocytopenia, and acute kidney injury (AKI) [1–4].

Gene abnormalities in complement-related factors in the alternative pathway or the production of anti-CFH autoantibodies can result in aHUS due to excess complement activation [2, 5–7]. These genetic abnormalities are found in approximately 40–60% of patients with aHUS [5–7]. In individuals with complement gene variants, the penetrance of aHUS has been reported to be 20–50% [8, 9]. Incomplete penetrance is common to all aHUS-associated complement genes, which suggests that the presence of a pathogenic variant alone is not sufficient for the development of the disease; the presence of a triggering stimulus is also necessary [8–12].

Previous studies reported poor outcomes with plasma therapy as the standard of care in patients with aHUS in the pre-eculizumab era [7, 8]. Plasma therapy resulted in end-stage kidney disease (ESKD) or death in 48% of paediatric and 67% of adult patients within 3 years of follow up [8]. In France, 56% of adult patients progressed to ESKD within the first year of aHUS, irrespective of the presence or absence or the type of complement dysregulation [7]. In contrast, the introduction of eculizumab (Alexion Pharmaceuticals, Boston, USA), a recombinant humanized monoclonal antibody to complement component C5, has changed the management of aHUS [13]. Eculizumab was approved for the treatment of aHUS in Japan in September 2013 [14]. Eculizumab prevents terminal complement activation by inhibiting the cleavage of C5 into C5a and C5b [14]. Regulatory-mandated post-marketing surveillance was conducted from September 2013 to July 2018 to assess the long-term safety and effectiveness of eculizumab in patients with aHUS in Japanese clinical practice, and an interim analysis, including the data sets of 29 adult patients with aHUS and 27 patients with secondary TMA, confirmed the safety and effectiveness of eculizumab [14]. Here, we report the findings from the full dataset analysis of the adult patients in the post-marketing surveillance. This study analysed 79 adult patients after exclusion of patients with TMA of complicated underlying diseases as secondary TMA, and compared patient characteristics and clinical courses between patients who did and did not meet effectiveness endpoints.

## Materials and methods

### Study design and patients

This analysis of the post-marketing surveillance includes the full dataset obtained from the patients enrolled by the end of January 2018. Adult patients (≥ 18 years) diagnosed with aHUS, who met the criteria in the Japanese aHUS clinical guide 2015, receiving ≥ 1 dose of eculizumab, were included in this analysis by the review of individual haematologic and renal variables and underlying diseases reported by an attending physician [1]. Per the guide, aHUS was diagnosed if MAHA, thrombocytopenia, and AKI were present, with the exclusion of Shiga toxin-producing *Escherichia coli*-HUS, thrombotic thrombocytopenic purpura (TTP) or secondary TMAs caused by complicated underlying diseases, i.e., autoimmune diseases, drugs, infection, malignant tumours, metabolic disorders, or transplantation [1]. If disintegrin-like and metalloproteinase with thrombospondin type 1 motifs 13 (ADAMTS13) activity was > 5 to 10%, TTP was excluded. According to the 2015 Japanese aHUS clinical guide, MAHA was defined as haemoglobin < 10 g/dL, elevated serum lactate dehydrogenase (LDH) levels, decrease of serum haptoglobin level, and presence of schistocytes on a peripheral blood smear to confirm [1]; thrombocytopenia, as platelet count (PLT) < 150 × 10^9^/L; and, AKI, per the Kidney Disease Improving Global Outcomes guideline [15].

Patient characteristics including demographics, disease characteristics, and genetic information on complement genes were recorded at initiation of eculizumab treatment (baseline). Patient data were collected at 6 months, 12 months, and annually thereafter. Patients who discontinued eculizumab (administration interval longer than 30 days) were observed until 12th July, 2018, pending agreement by the attending physician.

### Treatment

The dosing regimen per the approved label was as follows: 900 mg every week for the first 4 weeks, 1200 mg at week 5, and 1200 mg every 2 weeks thereafter. However, actual dosing and administration intervals were determined by the attending physician. Data on the duration of eculizumab administration, regimen used, and reason for discontinuation were collected. Before the first dose of eculizumab, anti-meningococcal vaccination was mandatory unless urgent treatment was required [16].

### Assessments of safety and effectiveness

The effectiveness endpoints of eculizumab were defined as shown in Supplementary Table S1. Patients with baseline parameters that met the endpoints were excluded in the analysis. For safety assessment, adverse events, serious adverse events and adverse drug reactions reported by the attending physician during the study were classified according to the Japanese translation of the Medical Dictionary for Regulatory Activities (Supplementary Table S1).

### Statistical analysis

In the safety analysis, the number of patients and incidence rates in patient-years for each event were calculated. Statistical significance of the changes from baseline in PLT, LDH, serum creatinine (sCr) and estimated glomerular filtration rate (eGFR) levels was evaluated by paired *t* test. The baseline data of patients who did or did not meet each endpoint were compared using Fisher’s exact test for categorical variables, and Wilcoxon rank sum test for continuous variables. Baseline factors and conditions of clinical practice were examined by stepwise multivariate logistic regression analysis in terms of the serum creatinine improvement from baseline or off-dialysis 90 days after eculizumab treatment, when improvement was observed in > 50% of patients. The time to achieve effectiveness endpoints was examined by Kaplan–Meier analysis. Missing data were not imputed. Statistical analyses were performed using SAS version 9.4 (SAS Institute, Cary, NC). Two-sided *p* values (significance level < 0.05) were used in all analyses.

## Results

### Patients

A total of 207 patients were diagnosed with aHUS as per the respective 2013 or 2015 clinical guides and received ≥ 1 dose of eculizumab from September 2013 to July 2018. Among them, 203 patients were enrolled in post-marketing surveillance. In this safety and effectiveness analysis, 79 adult patients were included as aHUS according to the 2015 clinical guide [1], after exclusion of 2 patients without any records at diagnosis, 2 patients who did not meet the criteria for TMA at diagnosis, and 42 patients with TMA complicated by underlying disease (Supplementary Fig. S1**)**.

### Baseline demographics and disease characteristics

At baseline, the median age (range) of patients was 54.0 (18–89) years. A family history of aHUS was reported by 3 (3.8%) patients. Complement gene variants were reported in 26 (50.0%) patients who were examined (Table 1, the detail of variants is shown in Supplementary Table S2). There were no records of anti-CFH antibody-positive patients among the 52 tested patients. The median duration (range) of eculizumab treatment was 30 (0–296) weeks; 42 patients (53.2%) received eculizumab treatment for ≥ 26 weeks (Table 1). Among them, 23 patients received more than one year of treatment (≥ 1 year, < 2 years; 9 patients, ≥ 2 years, < 3 years; 9 patients, ≥ 3 years; 5 patients).

**Table 1:** Baseline demographics and disease characteristics

Baseline demographics	Value
Median age at 1st eculizumab administration, years (range), *n* = 79	54.0 (18–89)
Median weight, kg (range), *n* = 77	53.0 (29.1–100.0)
Female sex, *n* (%)/total	43 (54.4)/79
Patient-reported family history of aHUS, *n* (%)/total	3 (3.8)/79
Patients with complement gene variants, *n* (%)/tested	26 (50)/52
One variant/polymorphism, *n* (%)/patients with variants in complement genes	19 (73.1)/26
Two or more variants/polymorphisms, *n* (%)/patients with variants in complement genes	7 (26.9)/26
*C3, n* (%)/patients with variants in complement genes	9 (34.6)/26
*CFB, n* (%)/patients with variants in complement genes	4 (15.4)/26
*CFH, n* (%)/patients with variants in complement genes	14 (53.8)/26
*CFHR1/3*^*a*^ deletion, *n* (%)/patients with variants in complement genes	0 (0.0)/26
*CFHR5, n* (%)/patients with variants in complement genes	1 (3.8)/26
*CFI, n* (%)/patients with variants in complement genes	2 (7.7)/26
*DGKE, n* (%)/patients with variants in complement genes	0 (0.0)/26
*MCP, n* (%)/patients with variants in complement genes	3 (11.5)/26
*THBD, n* (%)/patients with variants in complement genes	2 (7.7)/26
Pathogenic variants or VUS, *n* (%)/patients with variants in complement genes	20 (76.9)/26
Pathogenic variants, *n* (%)/patients with variants in complement genes	12 (46.2)/26
Anti CFH antibody, *n* (%)/patients tested	0 (0.0)/52
Clinical time course and pretreatment	
Median time from 1st TMA symptom to 1st administration of eculizumab, days (range), *n* = 73	19.0 (4–4652)
Median time from most recent TMA to plasma therapy, days (range), *n* = 67	4.0 (1–125)
Median time from most recent TMA to 1st administration of eculizumab, days (range), *n* = 76	17.5 (1–219)
Median time from most recent TMA to plasma therapy or 1st administration of eculizumab, days (range), *n* = 76	4.0 (1–125)
Median time from most recent TMA to diagnosis, days (range), *n* = 76	11.5 (1–132)
Median time from diagnosis to 1st administration of eculizumab, days (range), *n* = 78	2.5 (1–296)
Median days of plasma therapy from most recent TMA to 1st administration of eculizumab (range), *n* = 76	4.5 (0–26)
Plasma therapy (within 1 year before diagnosis), *n* (%)/total	51 (64.6)/79
Dialysis (during the 8 weeks before 1st administration of eculizumab), *n* (%)/total	50 (63.3)/79
Previous renal transplant, *n* (%)/total	2 (2.5)/79
Laboratory test values at baseline	
Median platelet count, × 10^9^/L, (range), *n* = 79	61.0 (3–558)
Platelet count ≤ 150 × 10^9^/L, *n* (%)/total	63 (79.7)/79
Median LDH level, U/L (range), *n* = 79	435.0 (140–7314)
LDH greater than ULN, *n* (%)/total	67 (84.8)/79
Median haemoglobin concentration, g/dL (range), *n* = 79	8.50 (3.9–13.5)
Haemoglobin concentration < 10 g/dL, *n* (%)/total	78 (98.7)/79
Median haptoglobin concentration, mg/dL (range), *n* = 65	10.0 (0–188)
Schistocytes positive, *n* (%)/examined	23 (95.8)/24
Median serum creatinine level, mg/dL (range), *n* = 78	3.08 (0.5–21.5)
Median eGFR, mL/min/1.73 m^2^ (range), *n* = 78	14.58 (2.1–82.6)
eGFR (mL/min/1.73 m^2^), *n*	78
< 15, *n* (%)	41 (52.6)
15–29, *n* (%)	17 (21.8)
30–44, *n* (%)	13 (16.7)
45–59, *n* (%)	2 (2.6)
60–89, *n* (%)	5 (6.4)
≥ 90, *n* (%)	0 (0.0)
Eculizumab treatment	
Median duration of eculizumab treatment, weeks (range), *n* = 79	30 (0–296)
< 1 week, *n* (%)	7 (8.9)
≥ 1 week, < 4 weeks, *n* (%)	17 (21.5)
≥ 4 weeks, < 26 weeks, *n* (%)	13 (16.5)
≥ 26 weeks, *n* (%)	42 (53.2)

*C3* complement component 3, *CFB* complement factor B, *CFH* complement factor H, *CFHR CFH*-related protein, *CFI* complement factor I, *DGKE* diacylglycerol kinase ε, *MCP* membrane cofactor protein, *THBD thrombomodulin. CFHR1/3* denotes the locus of *CFHR3* to *CFHR1* genes**.** *VUS* variant of unknown significance, *TMA* thrombotic microangiopathy, *LDH* lactate dehydrogenase, *ULN* upper limit of normal, *eGFR* estimated glomerular filtration rate

### Safety analysis in patients with aHUS

During eculizumab treatment, the total exposure time was 75.51 patient-years, and 94 serious adverse events (1.24 per patient-years) were reported in 39 out of 79 patients (Table 2). Among the serious adverse events, 40 adverse drug reactions (0.53 per patient-years) were reported in 20 patients (see footnote in Table 2 and Supplementary Table S3). The most frequently observed serious adverse event was renal impairment (9 events, 0.12 per patient-years). Three serious adverse events were reported 3 times each and involved pneumonia and TMA (3 events, 0.04 per patient-years (see the footnote in Table 2 for details), and 12 serious adverse events were reported twice. Adverse drug reactions were reported resulting in 2 serious adverse events, in 9 renal impairment events and in 3 pneumonia events. Among the serious adverse events of TMA, one occurred in a patient during eculizumab treatment at the approved dosage and intervals. The patient died due to sepsis (Patient #11 in Supplementary Table S4). Another TMA occurring before initiation of eculizumab treatment was not resolved in a patient with disseminated intravascular coagulation who received a single dose of eculizumab (Patient #12 in Supplementary Table S4). The third TMA-related event reported as an adverse drug reaction occurred during a period of extended dosing interval of 4 weeks (the patient did not meet discontinuation criteria), which appeared to be a recurrence and was fully resolved by reinstating approved dosing intervals. Other serious adverse events are listed in Supplementary Table S3. No meningococcal infections nor infusion reactions were reported in adult patients during eculizumab treatment. As described in Supplementary Table S4, there were 13 deaths during the post-marketing surveillance observation period, none of which were deemed by the attending physicians to be associated with eculizumab. Among the 13 deaths, including 5 patients in their 80’s, 7 patients died after one or 2 doses of eculizumab and 5 patients had adverse events considered as adverse drug reactions, in which the relationship between eculizumab and the adverse event was unknown or not ruled out.

**Table 2:** Most frequent serious adverse events (SAE)

Number of patients (*n*)	79	
Total exposure time (years)	75.51	
Total number of patients with at least one serious adverse event	39	
Total number of serious adverse events (patient-year)	94	(1.24)
SAE reported more than once	Number of SAEs	Patient-year
Renal impairment	9^a^	(0.12)
Pneumonia	3^a^	(0.04)
Thrombotic microangiopathy	3^b^	(0.04)
Cellulitis	2	(0.03)
Sepsis	2^b^	(0.03)
Pancytopenia	2^b^	(0.03)
Altered state of consciousness	2^a^	(0.03)
Cardiac failure	2^b^	(0.03)
Venous thrombosis limb	2^b^	(0.03)
Hypertension	2^b^	(0.03)
Secondary hypertension	2^b^	(0.03)
Respiratory failure	2	(0.03)
Enterocolitis	2^b^	(0.03)
Ascites	2^b^	(0.03)
Hepatic failure	2	(0.03)

Serious adverse events reported more than once are listed. Other serious adverse events reported once are listed in Supplementary Table S2

^a^Two SAEs were reported as adverse drug reactions (ADRs)

^b^One SAE was reported as an ADR

### Patient outcomes following eculizumab treatment

The levels of PLT, LDH, sCr and eGFR over time during eculizumab treatment are shown in Supplementary Fig. S2. All changes were statistically significant after 14 days of treatment with eculizumab. Thirty (43%) out of 70 patients who received dialysis at baseline discontinued it after a median (range) of 9.5 (1–464) days of treatment.

At the end of the observation period, complete TMA response (PLT normalization [≥ 150 × 10^9^/L], LDH normalization [≤ ULN], and ≥ 25% improvement in sCr from baseline for ≥ 4 weeks) was achieved by 18 out of 51 patients who had all the parameters needed for calculation and did not meet the parameters at baseline (35.3%, 95% CI 22.4–49.9) (Supplementary Table S5). The time to achievement of complete TMA response was examined by Kaplan–Meier analysis, which showed that 47.3% of patients were estimated to achieve the endpoint within 180 days (Supplementary Fig. S3). Early response of eculizumab treatment in patients, i.e., improvement of PLT (≥ 100 × 10^9^/L or an increase of ≥ 50% from baseline) and LDH (< 2 × ULN or a decrease of ≥ 50% from baseline), was also calculated by Kaplan–Meier analysis: Median time to improvement of PLT and LDH was 7 days and 12 days, respectively (Supplemental Fig. S4). Total survival of aHUS patients estimated by Kaplan–Meier analysis was 88.1% (Supplementary Fig. S5).

Of the 79 patients, 49 (62%) met the criteria for treatment discontinuation (administration interval longer than 30 days) by the end of the observation period; among them, 24 patients (49%; 24/49) discontinued treatment per the physician’s decision; 14 patients discontinued treatment because of sufficient response, symptom improvement, stable condition, or no recurrence following eculizumab treatment, and 5 patients discontinued due to no mutation in complement genes (Supplementary Table S6). No patients restarted eculizumab treatment due to TMA recurrence during the follow-up period.

### The difference in baseline characteristics between patient groups which did and did not meet effectiveness endpoints

The baseline characteristics of patients who did and did not meet complete TMA response and sCr improvement are summarized in Table 3 and Supplementary Table S7. The median duration of eculizumab treatment in patients who did not achieve complete TMA response and sCr improvement was 6 weeks and 5 weeks, respectively, which was considerably shorter than the corresponding duration in patients who did meet the endpoints, (114 weeks for complete TMA response, *p* < 0.00, and 43 weeks for sCr improvement, *p* < 0.001, respectively). A similar trend in median eculizumab treatment duration was found in patients meeting and not meeting the other endpoints, respectively (Supplementary Tables S8, S9). The presence/absence or the number (one/two or more) of complement gene variants did not significantly differ between the patients who did and did not meet those endpoints. There was no difference in patients with presence or absence of anti CFH antibodies.

**Table 3:** Comparison of clinical course and pre-treatment in the populations which did and did not achieve complete TMA response and serum creatinine level improvement

Clinical course and pretreatment	Complete TMA response (*n* = 51^a^)			Serum creatinine level improvement (*n* = 76^a^)		
	Patients met	Patients did not meet	*p* value	Patients met	Patients did not meet	*p* value
Median time from 1st TMA symptom to 1st administration of eculizumab, days (range)	18.0 (9–2852), *n* = 18	20.0 (4–4652), *n* = 28	0.821	17.0 (4–2852), *n* = 36	30.5 (5–4652), *n* = 34	0.020
Median time from most recent TMA to plasma therapy, days (range)	4.0 (1–13), *n* = 15	6.5 (1–125), *n* = 26	0.242	4.0 (1–13), *n* = 30	8.0 (1–125), *n* = 34	0.047
Median time from most recent TMA to 1st administration of eculizumab, days (range)	15.5 (1–31), *n* = 18	17.0 (1–165), *n* = 31	0.467	15.0 (1–61), *n* = 37	23.5 (2–219), *n* = 36	0.008
Medial time from the most recent TMA to plasma therapy or the 1st administration of eculizumab, days (range)	5.0 (1–24), *n* = 18	5.0 (1–125), *n* = 31	0.428	4.0 (1–28), *n* = 37	8.0 (1–125), *n* = 36	0.084
Median days of plasma therapy from most recent TMA to 1st administration of eculizumab (range)	2.0 (0–11), *n* = 18	4.0 (0–25), *n* = 31	0.606	2.0 (0–25), *n* = 37	6.0 (0–26), *n* = 36	0.048
Median time from diagnosis to 1st administration of eculizumab, days (range)	2.0 (1–8), n = 18	2.0 (1–41), *n* = 33	0.511	2.0 (1–296), *n* = 38	5 .0 (1–142), *n* = 37	0.069
Plasma therapy (within1 year before diagnosis), *n* (%)						
No	5 (27.8)	10 (30.3)	1.000	13 (33.3)	13 (35.1)	1.000
Yes	13 (72.2)	23 (69.7)		26 (66.7)	24 (64.9)	
Dialysis at diagnosis (within 1 year before diagnosis), *n* (%)						
No	6 (33.3)	17 (51.5)	0.250	16 (41.0)	20 (54.1)	0.358
Yes	12 (66.7)	16 (48.5)		23 (59.0)	17 (45.9)	
Median duration of eculizumab treatment, weeks (range)	114.0 (8–249), *n* = 18	6.0 (0–134), *n* = 33	< 0.001	43.0 (1–249), *n* = 39	5.0 (0–296), *n* = 37	< 0.001

*TMA* thrombotic microangiopathy, *aHUS* atypical haemolytic-uraemic syndrome, *LDH* lactate dehydrogenase, *eGFR* estimated glomerular filtration rate

^a^Data depict only patients for whom values were available for each assessed parameter. Patients with baseline parameters that met the endpoints were excluded in the analysis

**Fig. 1:**
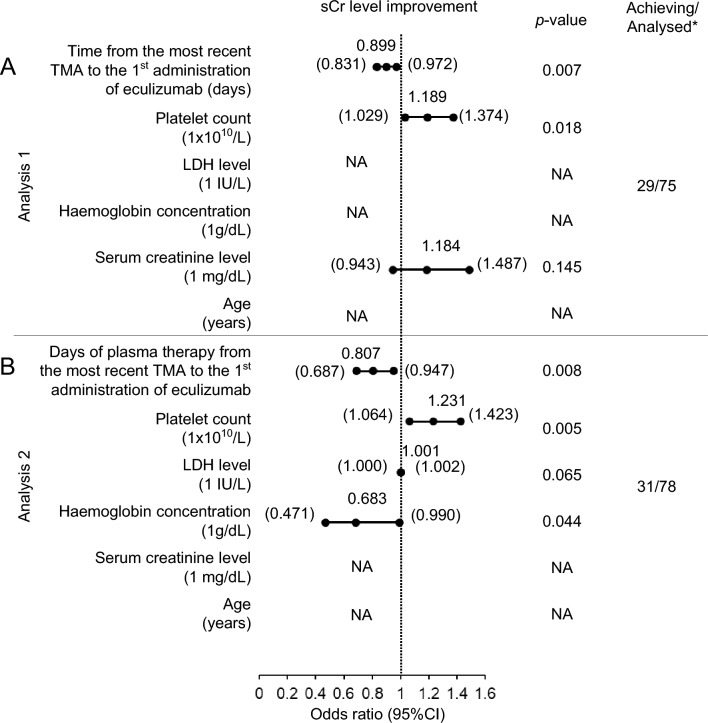
Stepwise multivariate logistic regression analysis on the improvement of renal function within 90 days after the 1st administration of eculizumab. The association of renal outcome with baseline laboratory parameters and treatment courses of **A** time from the most recent TMA to the 1st administration of eculizumab (analysis 1) or **B** days of plasma therapy (analysis 2) were examined by multivariate logistic regression analysis. The renal outcome was the improvement of > 25% sCr from baseline or off-dialysis by 90 days after the initiation of eculizumab. *sCr* serum creatinine, *CI* confidence interval, *NA* not applicable. *Number of patients. Data depict only those patients with values available for each assessed parameter

In patients who showed sCr level improvement, the median period from first TMA symptom (17.0 vs. 30.5 days, *p* = 0.020), from most recent TMA (15.0 vs. 23.5 days,* p* = 0.008), and of plasma therapy from most recent TMA (2.0 vs. 6.0 days, *p* = 0.048) to the first administration of eculizumab was shorter than the median period in patients who did not meet this endpoint (Table 3). Multivariate analysis of the factors at baseline affecting renal function improvement at 90 days after the 1st administration of eculizumab suggested that the time from the most recent TMA to eculizumab administration (OR 0.899, *p* = 0.007) and the days of plasma therapy (OR 0.807, *p* = 0.008) were negatively associated with the effectiveness endpoint (Fig. 1, Supplementary Fig. S6). Scatter plots for individual patients did not show statistically significant relationships between baseline characteristics, i.e., age, PLT, haemoglobin value, LDH level, sCr value or the presence of complement gene variants, and time from the most recent TMA to the 1st eculizumab administration (Supplementary Figs. S7–S12).

## Discussion

This data set analysis of the post-marketing surveillance for approximately 5 years evaluated the safety and effectiveness of eculizumab for the treatment of aHUS in adult patients in a real-world setting. This analysis also included a comparison of patient characteristics and clinical courses between patients who met effectiveness endpoints and those who did not, showing that renal outcomes were negatively associated with the time from the most recent TMA to the initiation of eculizumab treatment in clinical practice. Similar to the results from the interim analysis, no new safety signals were observed for adult patients with aHUS [14]. The interim analysis included 29 adult patients with aHUS with a median treatment duration of 24 weeks (range 1–103 weeks) [14]. The current study involved 79 adult patients with aHUS and a median treatment duration of 30 weeks (range 0–296 weeks).

In the safety analysis, no deaths related to eculizumab were recorded. The most frequently reported serious adverse event by the attending physician was renal impairment (9 events). Renal impairment might be a disease-related adverse event associated with aHUS, which was reported as an adverse event in the post-marketing surveillance. In addition, TMA was reported as a serious adverse event in 3 patients. In one of the cases, TMA may have been an aHUS recurrence, since it appeared during an extended dosing interval of 4 weeks and resolved after treatment with the approved dosing intervals. In other patients, the possibility of polymorphism C5 p.Arg885His (not examined in this cohort), which is present in ~ 3.2% of the Japanese population, was not excluded; this polymorphism prevents eculizumab from binding to C5, thereby blocking its therapeutic activity [17]. Since eculizumab prevents the terminal complement pathway through inhibition of C5 cleavage, and *Neisseria meningitidis* is primarily eliminated by the terminal complement components, patients treated with eculizumab are at increased risk of meningococcal infections [18]. Although no meningococcal infection was reported during this observation period, it is important to carefully manage the risk of meningococcal infection in patients treated with eculizumab.

Complete TMA response was achieved by 35% of patients who had all the parameters needed to calculate the complete TMA response; this result was consistent with a previous interim analysis, in which the percentage of patients was 28% [14]. This percentage was lower than that reported (65%) in a 26-week prospective clinical trial, enrolling patients with progressive TMA, which, based on patient inclusion criteria, would be similar to that in the post-marketing surveillance [18, 19]. However, patients with severe disease might be treated with eculizumab in real-world situations, which was suggested by laboratory test values at baseline. This trend, in which patients with severe disease were treated with eculizumab, has been previously described [20, 21]. Thus, the disparity in the achievement of the outcomes could be largely attributed to the differences between clinical and real-world studies, i.e., selected and unselected patient populations, and controlled or uncontrolled treatment regimens and duration.

According to our multivariate analysis, the time from the most recent TMA to the initiation of eculizumab was found to be inversely associated with the improvement of renal function. This result was similar to a post hoc analysis of pooled patients in clinical trials, which demonstrated that mean eGFR change from baseline at 1 year was significantly higher in patients treated within 7 days of onset than in those treated after 7 days, and that 81% and 47% of patients in the ≤ 7- and > 7-day groups, respectively, achieved a sustained increase in eGFR after 1 year [22]. Moreover, Brocklebank et al*.* also reported that shorter time between presentation and the first dose of eculizumab was associated with eGFR > 60 mL/min at 6 months [23]. The results of the current study and the recent study are evidence from real-world clinical practice, which suggests that early eculizumab initiation can lead to renal recovery in patients with aHUS.

In addition, the duration of plasma therapy was negatively associated with treatment effectiveness; however, shorter duration of plasma therapy might also contribute to the earlier initiation of eculizumab therapy. Previous studies reported poor outcomes with plasma therapy as the standard of care in patients with aHUS in the pre-eculizumab era [7, 8]. As described in guidelines on the use of therapeutic apheresis in clinical practice, “empiric plasma therapy, either as therapeutic plasma exchange or plasma infusion, is recommended while investigations for TTP and other forms of TMA are in progress or if eculizumab is not available. Once other causes of TMA have been excluded, eculizumab should be initiated [24, 25]. According to the Japanese guidelines, although plasma therapy allowed 83% of patients to achieve haematological remission, renal sequelae were reported in 80.3% of patients [23]. Additionally, in a Global aHUS registry analysis wherein 78.4% of patients with pregnancy-triggered aHUS and 70.9% of patients with aHUS not triggered by pregnancy were treated with plasma therapy, patients treated with eculizumab had a significantly lower risk of ESKD than those not treated with eculizumab [26]. Further evidence may be needed to optimize the treatment of patients with aHUS.

Limitations of this study include missing or underreporting of data, the different length of the observation period from patient to patient, and loss to follow-up. These limitations were largely inherent to the study design of the post-marketing surveillance, a real-world study, which can limit the generalizability of the findings from this analysis. In addition, patients were enrolled in the post-marketing surveillance as per diagnosis by the attending physician. We, however, highlight that post-marketing surveillance was mandated by the Japanese health authority to collect as much data as possible, and we have endeavoured to do so in this publication. This analysis focused on patients without TMA associated with other  underlying diseases, since the number of patients with each underlying disease was very small.

In conclusion, this post-marketing surveillance analysis in a real-world setting showed that renal outcomes were negatively associated with the time from the most recent TMA episode to the initiation of eculizumab treatment. No unexpected safety signals were identified in this population, and importantly, no instances of meningococcal infection were reported throughout the study period.

## Supplementary Information

Below is the link to the electronic supplementary material.

Supplementary file1 (DOC 781 KB)

## Data Availability

The data underlying this article are available in the article and in its online supplementary data. Further information may be requested from the corresponding authors.
