# Supplementary material for: Eculizumab for adult patients with atypical haemolytic-uraemic syndrome: full dataset analysis of Japanese post-marketing surveillance
Source: J Nephrol. 2024 May 29;37(8):2181–90. doi: 10.1007/s40620-024-01921-y (PMC11649742; doi:10.1007/s40620-024-01921-y)
Supplement: Supplementary file 1 — Supplementary file1 (DOC 781 KB) [file 40620_2024_1921_MOESM1_ESM.doc]

**Supplementary Materials to:**

**Eculizumab for adult patients with atypical haemolytic-uremic syndrome: Full dataset analysis of Japanese post-marketing surveillance**

**Maruyama S *et al*.**

**Supplementary Table S1.** Definition of clinical endpoints and adverse events

| Clinical endpoints of effectiveness | Definition |
| --- | --- |
| Complete thrombotic microangiopathy (TMA) response | Haematologic normalization and a ≥25% decrease in serum creatinine persisting for ≥4 weeks during eculizumab treatment* |
| Haematologic outcome |  |
| Haematologic normalization | PLT normalization (≥150×10^9^/L) and LDH normalization (less than upper limit of normal). Each normalization should be maintained for ≥4 weeks during eculizumab treatment* |
| Platelet count (PLT) normalization | ≥150×10^9^/L, maintained for ≥4 weeks during eculizumab treatment* |
| Lactic dehydrogenase (LDH) normalization | Less than upper limit of normal, which should be maintained for ≥4 weeks during eculizumab treatment* |
| Renal outcome |  |
| Serum creatinine level improvement | A decrease of ≥25% in serum creatinine should be maintained for ≥4 weeks during eculizumab treatment* |
| eGFR improvement | eGFR improvement of ≥15 mL/min/1.73 m^2^ should be maintained for ≥4 weeks during eculizumab treatment* |
| TMA event-free status | No decrease in PLT >25% after TMA remission (increase in PLT to ≥150×10^9^/L maintained for ≥2 weeks), no plasma exchange or fresh frozen plasma infusion, and no initiation of dialysis for ≥12 weeks during eculizumab treatment* |
| Adverse event | Definition |
| Adverse event (AE), serious AE (SAE) and adverse drug reaction (ADR) | Defined as any untoward symptoms, disease, and abnormal test values through the observational period irrespective of the causal relationship with eculizumab  Causal relationship between AEs and eculizumab was evaluated as “unrelated”, “probably related”, or “related” by an attending physician  An event was categorized as serious if it resulted in hospitalization, prolonged hospitalization, disability, permanent injury, or death, or was life-threatening  AE was considered as ADR if the causal relationship was other than “unrelated” and could not be denied. |

*The definition of endpoints was aligned with the definition in clinical trials [19].

**Supplementary Table S2.** Complement gene variants reported in patients

| Patient # | Variant | MAF | Note |
| --- | --- | --- | --- |
| 1 | CFH p.Arg1215Gly | (-) | pathogenic |
| 2 | C3 p.Ile1157Thr | (-) | pathogenic |
| 3 | C3 p.Ile1157Thr | (-) | pathogenic |
| 4 | CFH p.Val62Ile^†^ | 0.326  0.675 |  |
|  | CFH p.His402Tyr^†^ |  |  |
| 5 | MCP p.Ala311Val | 0.000566 | VUS |
| 6 | CFH c.3311-4T>A* | (-) | VUS |
| 7 | C3 p.Pro214Ser | (-) | pathogenic |
| 8 | CFH p.Ser1191Trp | (-) | pathogenic |
| 9 | C3 p.Arg1042Leu | (-) | pathogenic |
| 10 | CFH p.Arg1215Gly | (-) | pathogenic |
| 11 | CFH p.Glu936Asp^†^ | 0.199  0.0383  0.189  0.000743 | VUS |
|  | CFB p.Leu9His^†^ |  |  |
|  | THBD p.Ala473Val^†^ |  |  |
|  | CFHR5 p.Pro453Ser |  |  |
| 12 | C3 p.Ser182Pro | (-) | VUS |
| 13 | C3 p.Ile1157Thr | (-) | pathogenic |
| 14 | CFB p.Arg32Gln^†^ | 0.0976  0.326  0.675  0.199 |  |
|  | CFH p.Val62Ile^†^ |  |  |
|  | CFH p. HIs402Tyr^†^ |  |  |
|  | CFH p.Glu936Asp^†^ |  |  |
| 15 | CFH p.Arg232Gln | (-) | VUS |
| 16 | CFH p.His402Tyr^†^ | 0.675  0.199  (-) | pathogenic |
|  | CFH p.Glu936Asp^†^ |  |  |
|  | CFH p.Glu1198Asp |  |  |
| 17 | C3 p.Ile1157Thr | (-) | pathogenic |
| 18 | CFH p.His402Tyr^†^ | 0.675  0.199  0.016  (-) | VUS |
|  | CFH p.Glu936Asp^†^ |  |  |
|  | CFI p.Arg406His^†^ |  |  |
|  | CFB p.Thr388Ala* |  |  |
| 19 | CFH polymorphism^†^ |  | No detail of description, Haemolysis assay: 53% |
| 20 | CFB polymorphism^†^ |  | No detail of description |
|  | CFH polymorphism^†^ |  |  |
|  | CFI polymorphism^†^ |  |  |
| 21 | CFH p.Arg1215Gln | (-) | pathogenic |
| 22 | CFH p.His402Tyr^†^ | 0.675  0.199  0.189 |  |
|  | CFH p.Glu936Asp^†^ |  |  |
|  | THBD p.Ala473Val^†^ |  |  |
| 23 | MCP p.Ser13Phe | 0.00493 | VUS |
| 24 | C3 p.Ile1157Thr | (-) | pathogenic |
| 25 | C3 p.His16Gln* | 0.000483 | VUS |
| 26 | MCP |  | No detail of description |

C3, Complement component 3; CFB, complement factor B; CFH, complement factor H; MCP, membrane cofactor protein; THBD, thrombomodulin. MAF, minor allele frequency; VUS, Variant of unknown significance. *: Novel variant. †: Common variant

**Supplementary Table S3.** Serious adverse events reported once (0.01 per patient-year)

| Serious adverse event |
| --- |
| Clostridium difficile colitis* |
| Enterococcal infection* |
| Pneumonia staphylococcal* |
| β-haemolytic streptococcal infection* |
| Candida infection |
| Pneumocystis jirovecii pneumonia* |
| Infection |
| Device related infection* |
| Cytomegalovirus infection* |
| Viral rhinitis* |
| Bile duct cancer |
| Cytopenia* |
| Thrombocytopenia |
| Disseminated intravascular coagulation* |
| Immunosuppression* |
| Decreased appetite* |
| Hyperkalaemia |
| Cerebral artery occlusion |
| Cerebral infarction* |
| Thrombotic cerebral infarction |
| Posterior reversible encephalopathy syndrome |
| Epilepsy |
| Dementia* |
| Cardiac arrest |
| Ventricular tachycardia |
| Angina unstable |
| Cardiac failure congestive |
| Venous thrombosis* |
| Embolism |
| Peripheral artery thrombosis |
| Vena cava thrombosis |
| Haemorrhage* |
| Acute pulmonary oedema |
| Pleurisy* |
| Pulmonary haemorrhage |
| Upper respiratory tract inflammation* |
| Pancreatitis acute* |
| Haematochezia |
| Ileus paralytic |
| Gastrointestinal necrosis* |
| Retroperitoneal haemorrhage* |
| Cholecystitis |
| Cholelithiasis |
| Jaundice |
| Liver disorder |
| Urticaria* |
| Chronic kidney disease |
| Renal hypertension |
| Pyrexia |
| Multiple organ dysfunction syndrome* |
| Blood lactate dehydrogenase increased* |
| Haptoglobin decreased |
| Serum ferritin increased* |
| Vascular pseudoaneurysm |
| Shunt stenosis |
| * Serious adverse reaction reported as adverse drug reaction |

**Supplementary Table S4**. Description of the patients who died during PMS

| Patient # | Treatment duration and AE |
| --- | --- |
| 1* | Age 70s. Eculizumab was given for 184 days; the patient died for duodenal papilla cancer 20 days after last dose of eculizumab. Relationship between AE and eculizumab was denied. Complement gene testing was performed, but variant was not identified. |
| 2 | Age 10s. One dose of eculizumab was given; the patient died for respiratory failure 5 days after the administration of the unique dose of eculizumab. Relationship between AE and eculizumab was denied. Complement gene testing was not performed. |
| 3* | Age 30s. One dose of eculizumab was given; the patient died of cardiac arrest caused by myocardial conduction disturbances 3 days after the administration of the unique dose of eculizumab. Relationship between AE and eculizumab was denied. Complement gene testing was not performed. |
| 4 | Age 80s. Eculizumab was given for 350 days; the patient died for aortic aneurysm 177 days after last dose of eculizumab. Relationship between AE and eculizumab was denied. Complement gene testing was not performed. |
| 5* | Age 60s. Eculizumab was given for 43 days; the patient died for acute respiratory failure/heart failure caused by accidental ingestion 9 days after last dose of eculizumab. Relationship between AE and eculizumab was denied. Complement gene testing was not performed. |
| 6 | Age 80s. Eculizumab was given for 14 days; the patient died for hepatic failure 8 days after last dose of eculizumab. Relationship between AE and eculizumab was denied. Complement gene testing was not performed. |
| 7^†^ | Age 80s. Eculizumab was given for 280 days; the patient died for multiple organ dysfunction syndrome one day after last dose of eculizumab. Relationship between AE and eculizumab was unknown. Complement gene testing was performed, but variant was not identified. |
| 8^†^ | Age 80s. Eculizumab was given for 55 days; the patient died for bacterial pneumonia 384 days after last dose of eculizumab. Relationship between AE and eculizumab was unknown. Complement gene testing was not performed. |
| 9^†^ | Age 30s. Eculizumab was given for 14 days; the patient died for the unresolved TMA from the onset 14 days after last dose of eculizumab. Relationship between AE and eculizumab was unknown. Complement gene testing was not performed. |
| 10 | Age 80s. Eculizumab was given for 7 days; the patient died for heart failure and respiratory failure one day after last dose of eculizumab. Relationship between AE and eculizumab was denied. Complement gene testing was not performed. |
| 11^†^ | Age 30s. Eculizumab was given for 233 days; the patient died for pulmonary haemorrhage caused by sepsis 3 days after last dose of eculizumab. Relationship between AE and eculizumab was not denied. Complement gene variants were identified (CFH p.His402Tyr, CFH p.Glu936Asp, THBD p.Ala473Val). |
| 12 | Age 70s. One dose of eculizumab was given; the patient died for multiple organ failure caused by TMA 10 days after last dose of eculizumab. Relationship between AE and eculizumab was denied. Complement gene testing was not performed. |
| 13^†^ | Age 50s. Eculizumab was given for 7 days; the patient died for gastrointestinal necrosis and retroperitoneal haemorrhage 4 days after last dose of eculizumab. Relationship between AE and eculizumab was not denied. Complement gene testing was not performed. |
| * Reported in a report for interim analysis (14). ^†^ AE was considered as ADR, in which the relationship between eculizumab and AE was unknown or not denied. | |

**Supplementary Table S5.** Effectiveness of eculizumab in adult patients with aHUS at the end of observation.

| Effectiveness endpoints (n*) | Number of patients achieving | % | 95%CI |
| --- | --- | --- | --- |
| Complete TMA response (51) | 18 | 35.3 | 22.4 - 49.9 |
| Haematologic normalization (52) | 21 | 40.4 | 27.0 - 54.9 |
| Platelet count normalization (61) | 30 | 49.2 | 36.1 - 62.3 |
| LDH normalization (66) | 33 | 50.0 | 37.4 - 62.6 |
| Serum creatinine level improvement (76) | 39 | 51.3 | 39.6 - 63.0 |
| eGFR improvement (76) | 27 | 35.5 | 24.9 – 47.3 |
| TMA event-free status (78) | 39 | 50.0 | 38.5 - 61.5 |

aHUS, atypical haemolytic-uremic syndrome; CI, confidence interval; TMA, thrombotic microangiopathy; LDH, lactate dehydrogenase; eGFR, estimated glomerular filtration rate. *: Data depict only those patients with values available for each assessed parameter. Patients with baseline parameters that met the endpoints were excluded in the analysis.

**Supplementary Table S6**. Reasons/situations of treatment discontinuation

| Administration status | | | All patients |
| --- | --- | --- | --- |
|  |  |  |  |
| Number of patients | | | 79 |
| Continued | | | 30 (38.0) |
| Discontinued (including overlapped patients) | | | 49 (62.0) |
|  | Doctor’s decision* | | 24 (49.0) |
|  |  | Sufficient response/symptom improvement/stable condition/no recurrence | 14 (58.3) |
|  |  | Insufficient response | 1 (4.2) |
|  |  | Change diagnosis to other than aHUS | 3 (12.5) |
|  |  | No mutation in complement genes | 5 (20.8) |
|  |  | Changing hospital | 0 (0.0) |
|  |  | Others | 1 (4.2) |
|  | Insufficient response* | | 10 (20.4) |
|  |  | No improvement of renal function | 7 (70.0) |
|  |  | No improvement of haemolysis | 5 (50.0) |
|  |  | No improvement of platelet count | 7 (70.0) |
|  |  | Others | 1 (10.0) |
|  | Patient or caregiver request | | 5 (10.2) |
|  | Adverse event | | 6 (12.2) |
|  | Pregnancy | | 0 (0.0) |
|  | Death | | 10 (20.4) |
|  | Sufficient response/symptom improvement/stable condition/no recurrence | | 0 (0.0) |
|  | Change diagnosis to other than aHUS | | 0 (0.0) |
|  | No mutation in complement genes | | 0 (0.0) |
|  | Changing hospital | | 1 (2.0) |
|  | Others | | 0 (0.0) |

* The number of patients are overlapping.

**Supplementary Table S7.** Comparison of baseline patient characteristics in the populations which met and did not meet complete TMA response and serum creatinine level improvement

| Baseline demographics and laboratory test value at baseline | | | Complete TMA response (n=51*) | | | Serum creatinine level improvement (n=76*) | | |
| --- | --- | --- | --- | --- | --- | --- | --- | --- |
|  |  |  | Patients met | Patients did not meet | *p-value* | Patients met | Patients did not meet | *p-value* |
| Median age at 1^st^ eculizumab administration, years (range) | | | 42.5 (19-73),  n=18 | 54 (18-86),  n=33 | 0.214 | 48.0 (19-86),  n=39 | 58.0 (18-89),  n=37 | 0.349 |
| Median Weight, kg (range) | | | 53.95 (38.8-83.3), n=18 | 52.35 (37.1-100.0), n=32 | 0.863 | 52.10 (38.8-100), n=39 | 54.00 (29.1-79.6), n=35 | 0.488 |
| Sex, n (%) | Female | | 12 (66.7) | 17 (51.5) | 0.380 | 22 (56.4) | 18 (48.6) | 0.646 |
|  | Male | | 6 (33.3) | 16 (48.5) |  | 17 (43.6) | 19 (51.4) |  |
| Patient reported family history of aHUS n, (%) | Unknown | | 1 (5.6) | 4 (12.1) | 1.000 | 2 (5.1) | 4 (10.8) | 0.241 |
|  | No | | 16 (88.9) | 28 (84.8) |  | 34 (87.2) | 33 (89.2) |  |
|  | Yes | | 1 (5.6) | 1 (3.0) |  | 3 (7.7) | 0 (0.00) |  |
| Patients who have variants in complement genes, n (%) | Unknown | | 4 (22.2) | 15 (45.5) | 0.724 | 12 (30.8) | 18 (48.6) | 0.370 |
|  | No | | 5 (27.8) | 8 (24.2) |  | 10 (25.6) | 10 (27.0) |  |
|  | Yes | | 9 (50.0) | 10 (30.3) |  | 17 (43.6) | 9 (24.3) |  |
| One variant/polymorphism, n (%) | | | 7 (38.9) | 6 (18.2) | 0.628 | 14 (35.9) | 5 (13.5) | 0.188 |
| Two or more variants/polymorphisms, n (%) | | | 2 (11.1) | 4 (12.1) |  | 3 (7.7) | 4 (10.8) |  |
| Pathogenic/variant of unknown significance, n (%) | | No | 1 (5.6) | 3 (9.1) | 0.582 | 3 (7.7) | 3 (8.1) | 0.627 |
|  |  | Yes | 8 (44.4) | 7 (21.2) |  | 14 (35.9) | 6 (16.2) |  |
| Median platelet count, x10^9^/L (range) | | | 67.0 (6-149), n=18 | 33.0 (3-113), n=33 | 0.055 | 81.0 (3-558), n=39 | 49.0 (5-269), n=37 | 0.234 |
| Median LDH level, U/l (range) | | | 764.5 (234-5915), n=18 | 578.0 (287-4989), n=33 | 1.000 | 578.0 (161-7314), n=39 | 379.0 (140-3882), n=37 | 0.086 |
| Median haemoglobin concentration, g/dl (range) | | | 7.15 (3.9-13.1), n=18 | 8.50 (5.8-13.5), n=33 | 0.183 | 7.70 (3.9-13.1), n=39 | 8.70 (5.8-13.5), n=37 | 0.174 |
| Median haptoglobin concentration, mg/dl (range) | | | 10.0 (1-10), n=14 | 10 (0-121), n=29 | 0.626 | 10 (1-188), n=29 | 10 (0-121), n=33 | 0.348 |
| Median serum creatinine level, mg/dl (range) | | | 3.08 (1.3-21.5), n=18 | 3.17 (0.6-9.9), n=33 | 0.509 | 3.59 (1.2-21.5), n=39 | 2.63 (0.5-9.9), n=37 | 0.050 |
| Median eGFR, ml/min/1.73 m^2^ (range) | | | 14.07 (2.1-62.0), n=18 | 16.11 (4.7-78.0), n=33 | 0.349 | 12.57 (2.1-62.0), n=39 | 16.58 (4.7-82.6), n=37 | 0.034 |

*: Data depict only those patients with values available for each assessed parameter. Patients with baseline parameters that met the endpoints were excluded in the analysis.

**Supplementary Table S8.** Comparison of baseline patient characteristics in the populations which met and did not meet haematologic normalization and platelet count normalization

| Items | | Haematologic normalization (n=52*) | | | Platelet count normalization (n=61*) | | |
| --- | --- | --- | --- | --- | --- | --- | --- |
|  |  | Patients met | Patients did not meet | *p-value* | Patients met | Patients did not meet | *p-value* |
| Median age at 1^st^ eculizumab administration, years (range) | | 42.0 (19-73),  n=21 | 58.0 (18-86),  n=31 | 0.037 | 44.0 (19-80), n=30 | 58.0 (18-89), n=31 | 0.184 |
| Median Weight, kg (range) | | 53.95 (38.8-83.3), n=20 | 53 .0 (37.1-100.0), n=31 | 0.915 | 52.10 (37.1-83.3), n=29 | 54.00 (29.1-100), n=30 | 0.897 |
| Sex, n (%) | Female | 13 (61.9) | 17 (54.8) | 0.775 | 19 (63.3) | 15 (48.4) | 0.305 |
|  | Male | 8 (38.1) | 14 (45.2) |  | 11 (36.7) | 16 (51.6) |  |
| Patient reported family history of aHUS, n (%) | Unknown | 1 (4.8) | 4 (12.9) | 1.000 | 2 (6.7) | 3 (9.7) | 0.490 |
|  | No | 19 (90.5) | 26 (83.9) |  | 26 (86.7) | 28 (90.3) |  |
|  | Yes | 1 (4.8) | 1 (3.2) |  | 2 (6.7) | 0 (0.00) |  |
| Patients who have variants in complement genes, n (%) | Unknown | 5 (23.8) | 15 (48.4) | 1.000 | 10 (33.3) | 14 (45.2) | 0.517 |
|  | No | 6 (28.6) | 7 (22.6) |  | 8 (26.7) | 9 (29.0) |  |
|  | Yes | 10 (47.6) | 9 (29.0) |  | 12 (40.0) | 8 (25.8) |  |
| One variant/polymorphism, n (%) | | 7 (33.3) | 6 (19.4) | 1.000 | 8 (26.7) | 5 (16.1) | 1.000 |
| Two or more variants/polymorphisms, n (%) | | 3 (14.3) | 3 (9.7) |  | 4 (13.3) | 3 (9.7) |  |
| Pathogenic/variant of unknown significance, n (%) | No | 1 (4.8) | 3 (9.7) | 0.303 | 2 (6.7) | 3 (9.7) | 0.347 |
|  | Yes | 9 (42.9) | 6 (19.4) |  | 10 (33.3) | 5 (16.1) |  |
| Median time from 1^st^ TMA symptom to the 1^st^ administration of eculizumab, days (range) | | 18.0 (9-2852), n=21 | 17.5 (4-4652), n=26 | 0.707 | 18.5 (4-2852), n=30 | 18.5 (7-4652), n=26 | 0.980 |
| Median time from the most recent TMA to plasma therapy, days (range) | | 4.0 (1-30), n=18 | 6.5 (1-125), n=24 | 0.338 | 4.0 (1-74), n=26 | 5.0 (1-125), n=25 | 0.629 |
| Median time from the most recent TMA to the 1^st^ administration of eculizumab, days (range) | | 17.0 (1-43), n=21 | 15.0 (1-165), n=29 | 0.952 | 17.5 (1-103), n=30 | 16.0 (1-219), n=29 | 0.969 |
| Median time from the most recent TMA to plasma therapy or to the 1^st^ administration of eculizumab, days (range) | | 4.0 (1-30), n=21 | 5.0 (1-125), n=29 | 0.553 | 4.0 (1-74), n=30 | 5.0 (1-125), n=29 | 0.789 |
| Median time from the day of diagnosis to the 1^st^ administration of eculizumab, days (range) | | 2.0 (1-30), n=21 | 2.0 (1-41), n=31 | 0.704 | 2.0 (1-30), n=30 | 2.0 (1-88), n=31 | 0.419 |
| Median days of plasma therapy from the most recent TMA to the 1^st^ administration of eculizumab (range) | | 3.0 (0-25), n=21 | 4.0 (0-20), n=29 | 0968 | 4.0 (0-25), n=30 | 4.0 (0-20), n=29 | 0.842 |
| Plasma therapy (past 1 year before diagnosis), (%) | No (%) | 5 (23.8) | 10 (32.3) | 0.551 | 7 (23.3) | 13 (41.9) | 0.173 |
|  | Yes (%) | 16 (76.2) | 21 (67.7) |  | 23 (76.7) | 18 (58.1) |  |
| Dialysis at diagnosis (past 1 year before diagnosis), (%) | No (%) | 8 (38.1) | 16 (51.6) | 0.402 | 11 (36.7) | 19 (61.3) | 0.074 |
|  | Yes (%) | 13 (61.9) | 15 (48.4) |  | 19 (63.3) | 12 (38.7) |  |
| Median platelet count, x10^9^/L, (range) | | 68 (6-149), n=21 | 27 (3-108), n=31 | 0.005 | 70 (3-149), n=30 | 27 (5-104), n=31 | <0.001 |
| Median LDH level, U/l (range) | | 755.0 (234-5915), n=21 | 578.0 (287-4989), n=31 | 0.852 | 719.5 (187-5915), n=30 | 446.0 (179-3882), n=31 | 0.382 |
| Median haemoglobin concentration, g/dl (range) | | 7.40 (3.9-13.1), n=21 | 8.00 (5.8-13.5), n=31 | 0.702 | 8.30 (3.9-13.1), n=30 | 7.90 (5.8-13.5), n=31 | 0.783 |
| Median haptoglobin concentration, mg/dl (range) | | 10 (1-10), n=17 | 10 (0-121), n=27 | 0.541 | 10 (1-67), n=26 | 10 (0-121), n=26 | 0.503 |
| Median serum creatinine level, mg/dl (range) | | 3.06 (1.0-21.5), n=21 | 3.29 (0.6-9.9), n=30 | 0.766 | 3.09 (1.0-21.5), n=30 | 2.57 (0.5-9.9), n=30 | 0.153 |
| Median eGFR, ml/min/1.73 m^2^ (range) | | 14.09 (2.1-78.0), n=21 | 15.80 (4.7-73.7), n=30 | 0.639 | 13.97 (2.1-78.0), n=30 | 18.82 (4.7-82.6), n=30 | 0.079 |
| Median duration of eculizumab treatment, weeks (range) | | 112.0 (8-249), n=21 | 5.0 (0-57), n=31 | <0.001 | 48.0 (3-249), n=30 | 3.0 (0-57), n=31 | <0.001 |

*: Data depict only those patients with values available for each assessed parameter. Patients with baseline parameters that met the endpoints were excluded in the analysis.

**Supplementary Table S9.** Comparison of baseline patient characteristics in the populations which met and did not meet LDH normalization and TMA event free status

| **Items** | | LDH normalization (n=66*) | | | TMA event free status (n=78*) | | |
| --- | --- | --- | --- | --- | --- | --- | --- |
|  |  | Patients met | Patients did not meet | *p-value* | Patients met | Patients did not meet | *p-value* |
| Median age at 1^st^ eculizumab administration, years (range) | | 44.0 (19-80), n=33 | 55.0 (18-86), n=33 | 0.383 | 49.0 (24-80),  n=39 | 58.0 (18-89),  n=39 | 0.988 |
| Median Weight, kg (range) | | 53.80 (38.8-83.3), n=32 | 49.0 (37.1-100), n=33 | 0.382 | 51.85 (37.1-76.3), n=38 | 55.55 (29.1-100.0), n=38 | 0.212 |
| Sex, n (%) | Female | 18 (54.5) | 19 (57.6) | 1.000 | 25 (64.1) | 17 (43.6) | 0.111 |
|  | Male | 15 (45.5) | 14 (42.4) |  | 14 (35.9) | 22 (56.4) |  |
| Patient reported family history of aHUS, n (%) | Unknown | 3 (9.1) | 3 (9.1) | 1.000 | 3 (7.7) | 3 (7.7) | 1.000 |
|  | No | 28 (84.8) | 29 (87.9) |  | 34 (87.2) | 35 (89.7) |  |
|  | Yes | 2 (6.1) | 1 (3.0) |  | 2 (5.1) | 1 (2.6) |  |
| Patients who have variants in complement genes, n (%) | Unknown | 9 (27.3) | 16 (48.5) | 1.000 | 14 (35.9) | 17 (43.6) | 1.000 |
|  | No | 10 (30.3) | 7 (21.2) |  | 11 (28.2) | 10 (25.6) |  |
|  | Yes | 14 (42.4) | 10 (30.3) |  | 14 (35.9) | 12 (30.8) |  |
| One variant/polymorphism, n (%) | | 11 (33.3) | 7 (21.2) | 0.665 | 11 (28.2) | 8 (20.5) | 0.665 |
| Two or more variants/polymorphisms, n (%) | | 3 (9.1) | 3 (9.1) |  | 3 (7.7) | 4 (10.3) |  |
| Pathogenic/variant of unknown significance, n (%) | No | 2 (6.1) | 3 (9.1) | 0.614 | 2 (5.1) | 4 (10.3) | 0.365 |
|  | Yes | 12 (36.4) | 7 (21.2) |  | 12 (30.8) | 8 (20.5) |  |
| Median time from 1^st^ TMA symptom to the 1^st^ administration of eculizumab, days (range) | | 18.5 (6-2852), n=32 | 15.5 (4-4652), n=28 | 0.279 | 19.0 (5-227), n=38 | 18.5 (4-4652), n=34 | 0.901 |
| Median time from the most recent TMA to plasma therapy, days (range) | | 4.0 (1-125), n=29 | 4.0 (1-74), n=25 | 0.807 | 4.0 (1-74), n=36 | 6.5 (1-125), n=30 | 0.171 |
| Median time from the most recent TMA to the 1^st^ administration of eculizumab, days (range) | | 18.0 (1-165), n=32 | 13.0 (1-103), n=31 | 0.308 | 18.5 (2-142), n=38 | 15.0 (1-219), n=37 | 0.370 |
| Median time from the most recent TMA to plasma therapy or to the 1^st^ administration of eculizumab, days (range) | | 4.5 (1-125), n=32 | 4.0 (1-74), n=31 | 0.977 | 4.0 (1-74), n=38 | 4.0 (1-125), n=37 | 0.455 |
| Median time from the day of diagnosis to the 1^st^ administration of eculizumab, days (range) | | 2.0 (1-296), n=32 | 2.0 (1-47), n=33 | 0.136 | 2.5 (1-296), n=38 | 2.0 (1-88), n=39 | 0.892 |
| Median days of plasma therapy from the most recent TMA to the 1^st^ administration of eculizumab (range) | | 6.0 (0-25), n=32 | 3.0 (0-20), n=31 | 0.237 | 6.0 (0-26), n=38 | 4.0 (0-20), n=37 | 0.199 |
| Plasma therapy (past 1 year before diagnosis), (%) | No (%) | 10 (30.3) | 12 (36.4) | 0.794 | 8 (20.5) | 19 (48.7) | 0.016 |
|  | Yes (%) | 23 (69.7) | 21 (63.6) |  | 31 (79.5) | 20 (51.3) |  |
| Dialysis at diagnosis (past 1 year before diagnosis), (%) | No (%) | 12 (36.4) | 18 (54.5) | 0.216 | 13 (33.3) | 25 (64.1) | 0.012 |
|  | Yes (%) | 21 (63.6) | 15 (45.5) |  | 26 (66.7) | 14 (35.9) |  |
| Median platelet count, x10^9^/L, (range) | | 81.0 (6-252), n=33 | 40.0 (3-558), n=33 | 0.023 | 111.0 (6-269), n=39 | 33.0 (3-558), n=39 | <0.001 |
| Median LDH level, U/l (range) | | 363.0 (234-5915), n=33 | 578.0 (252-7314), n=33 | 0.029 | 363.0 (140-5915), n=39 | 477.0 (179-7314), n=39 | 0.357 |
| Median haemoglobin concentration, g/dl (range) | | 8.40 (3.9-13.1), n=33 | 8.50 (5.6-13.5), n=33 | 0.984 | 8.50 (3.9-11.8), n=39 | 8.50 (5.6-13.5), n=39 | 0.645 |
| Median haptoglobin concentration, mg/dl (range) | | 10 (1-72), n=26 | 10 (0-188), n=27 | 0.849 | 10 (1-188), n=31 | 10 (0-121), n=33 | 0.583 |
| Median serum creatinine level, mg/dl (range) | | 3.06 (1.0-21.5), n=33 | 3.94 (0.6-9.9), n=32 | 0.900 | 3.09 (1.0-21.5), n=39 | 3.09 (0.5-9.9), n=38 | 0.315 |
| Median eGFR, ml/min/1.73 m^2^ (range) | | 14.26 (2.1-78.0), n=33 | 12.48 (4.7-73.7), n=32 | 0.952 | 14.06 (2.1-78.0), n=39 | 17.34 (4.7-82.6), n=38 | 0.108 |
| Median duration of eculizumab treatment, weeks (range) | | 71.0 (6-249), n=33 | 3.0 (0-115), n=33 | <0.001 | 56.0 (16-296), n=39 | 3.0 (0-115),  n=39 | <0.001 |

*: Data depict only those patients with values available for each assessed parameter. Patients with baseline parameters that met the endpoints were excluded in the analysis.

**
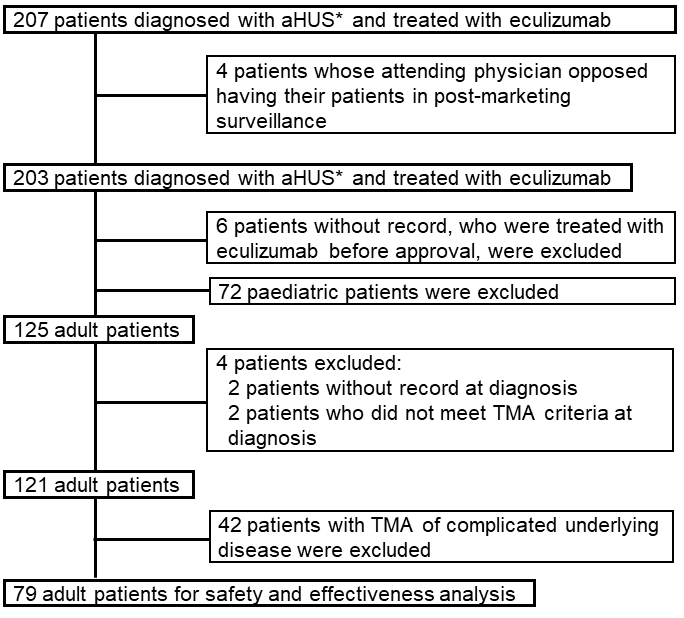
**

**Supplementary Fig. S1. Patient disposition**

Two-hundred three patients were diagnosed with aHUS based on Japanese aHUS clinical guide 2013 or 2015 and enrolled in post-marketing surveillance (*). Seventy-nine adult patients were subjects of this analysis according to Japanese aHUS clinical guide 2015. aHUS, atypical haemolytic uremic syndrome: TMA, thrombotic microangiopathy. For effectiveness analysis, patients with baseline parameters that met the endpoints were excluded in the analysis.

**
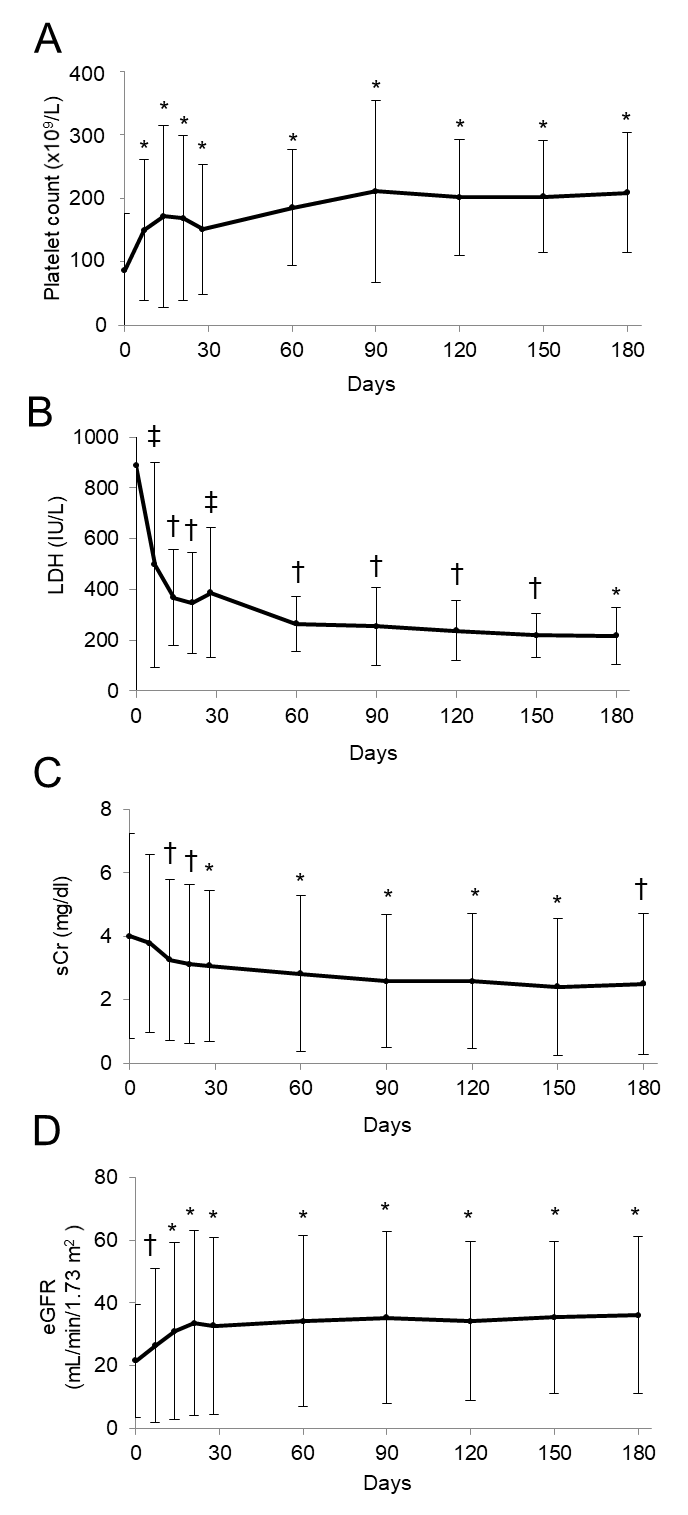
**

**Supplementary Fig. S2. Changes in haematologic and renal outcomes following eculizumab treatment**

Changes in platelet count (A), LDH (B), sCr (C) and eGFR (D) over time following initiation of eculizumab treatment (starting at day 0) are shown. All changes were statistically compared to the baseline (*, *p*<0.001; †, *p*<0.01; ‡; *p*<0.05). LDH, lactate dehydrogenase; sCr, serum creatinine; eGFR, estimated glomerular filtration rate.

**
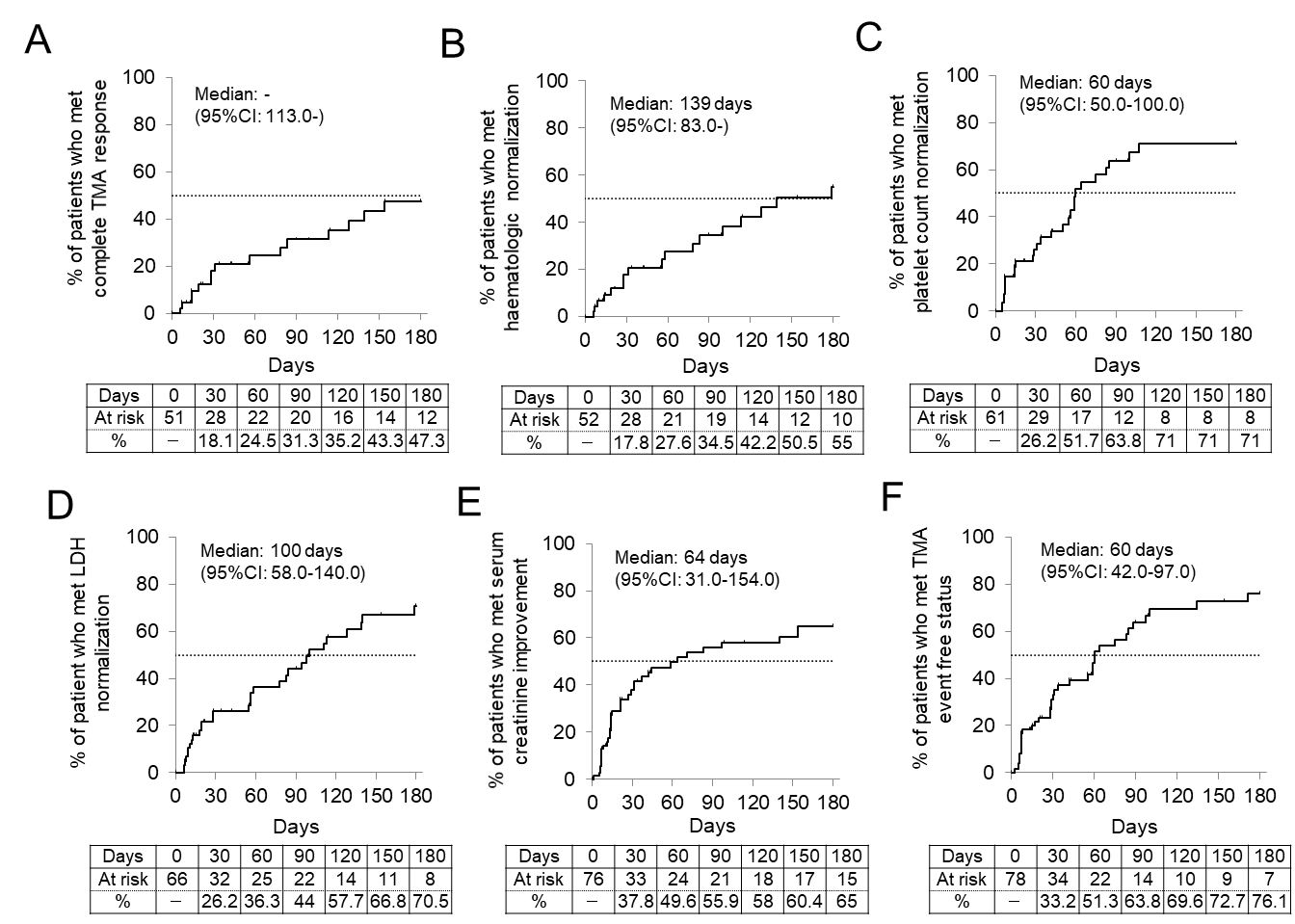
**

**Supplementary Fig. S3. Time to achieve effectiveness endpoints**

(A-F) The percentage of patients achieving complete TMA response (A), haematologic normalization (B), platelet count (C), LDH normalization (D), serum creatinine (E), and TMA event-free status (F) was estimated using Kaplan–Meier analysis. TMA, thrombotic microangiopathy; CI, confidence interval; LDH, lactate dehydrogenase.


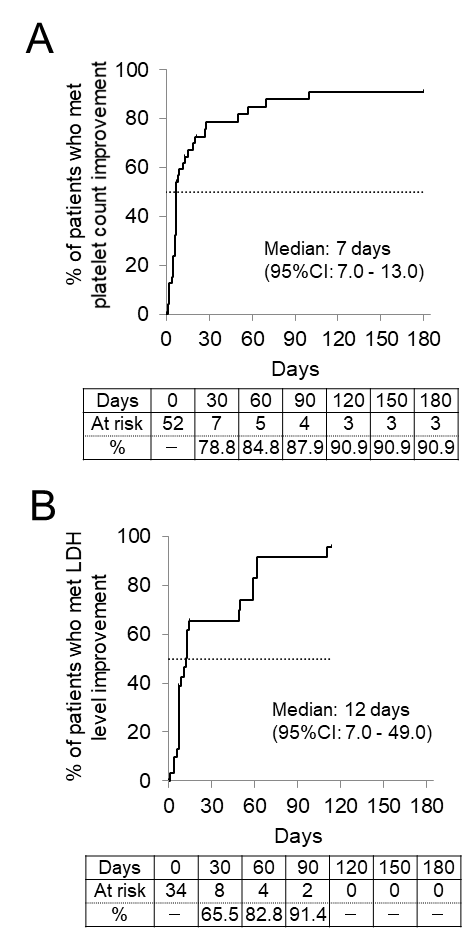


**Supplementary Fig. S4. Time to improve platelet count and LDH level**

Improvement of PLT (≥100× 109/L or an increase of ≥50% from baseline) (A) and LDH (<2xULN or a decrease of ≥50% from baseline) (B) were calculated by Kaplan–Meier analysis as early responses of eculizumab treatment in patients. TMA, thrombotic microangiopathy; CI, confidence interval; LDH, lactate dehydrogenase.

**
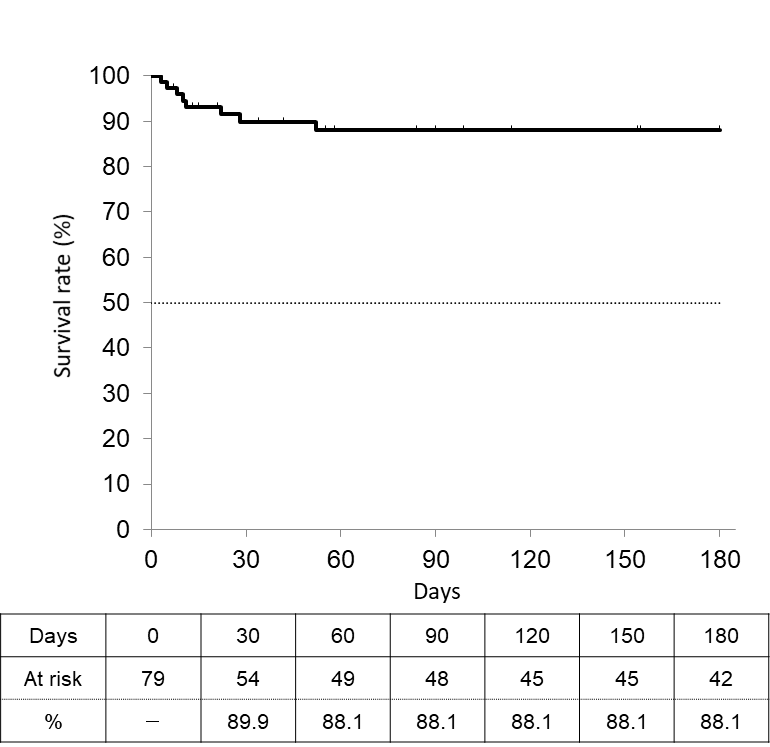
**

**Supplementary Fig. S5.** **Overall survival of patients with aHUS treated with eculizumab**

Kaplan–Meier analysis was used to calculate time-to-event values for overall survival from the date of 1^st^ administration of eculizumab to the date of occurrence of the events.


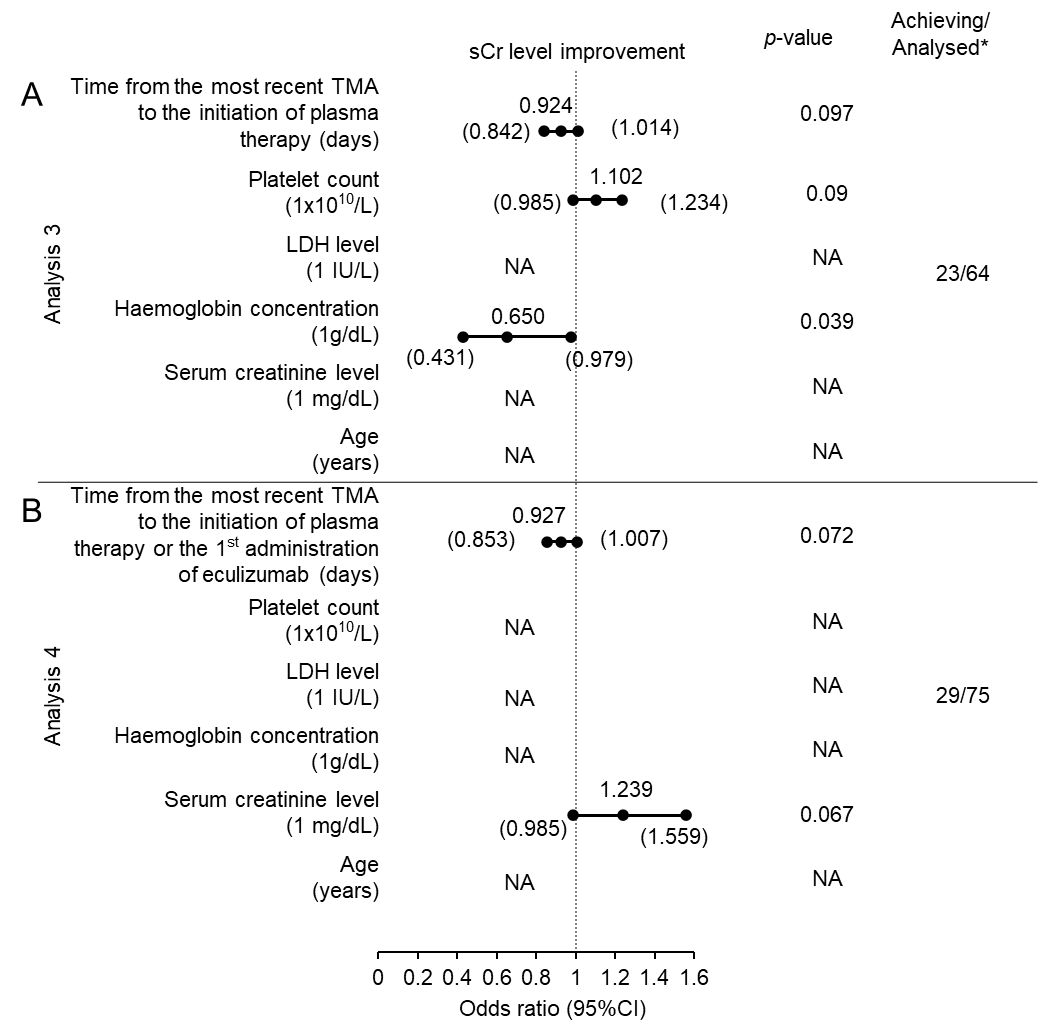


**Supplementary Fig. S6. Stepwise multivariate logistic regression analysis on the improvement of renal function by 90 days after 1^st^ administration of eculizumab**

The association of renal outcome with baseline laboratory parameters and treatment courses of (A) time from the most recent TMA to the initiation of plasma therapy (analysis 3) or (B) time from the most recent TMA to the initiation of plasma therapy or the 1^st^ administration of eculizumab (analysis 4) were examined by multivariate logistic regression analysis. The renal outcome was the improvement of >25% sCr from baseline or off-dialysis by 90 days after the initiation of eculizumab. sCr, serum creatinine; CI, confidence interval; NA, not applicable; TMA, thrombotic microangiopathy. *, number of patients.

**
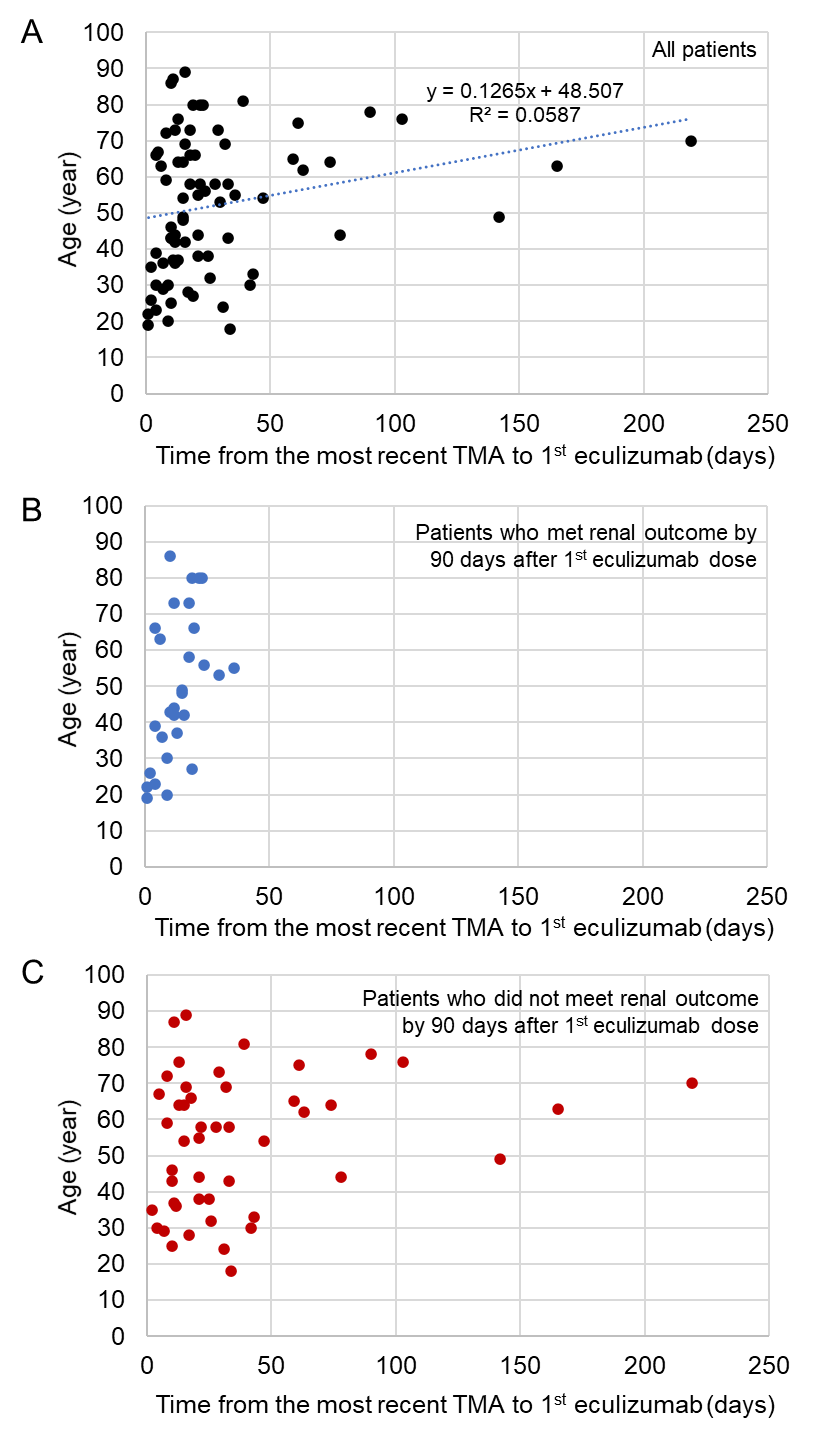
**

**Supplementary Fig. S7.** **Scatter plots of individual patients showing their ages for time from the most recent TMA to 1^st^ eculizumab**

Scatter plots of individual patients of all (A), who met improvement of renal function (>25% sCr from baseline or off-dialysis by 90 days after the initiation of eculizumab) (B) and, who did not meet the renal outcome (C) were generated to show relationship between ages at baseline and time from the most recent TMA to 1^st^ eculizumab. Approximate curve was generated to identify R^2^ in A. TMA, thrombotic microangiopathy.

**
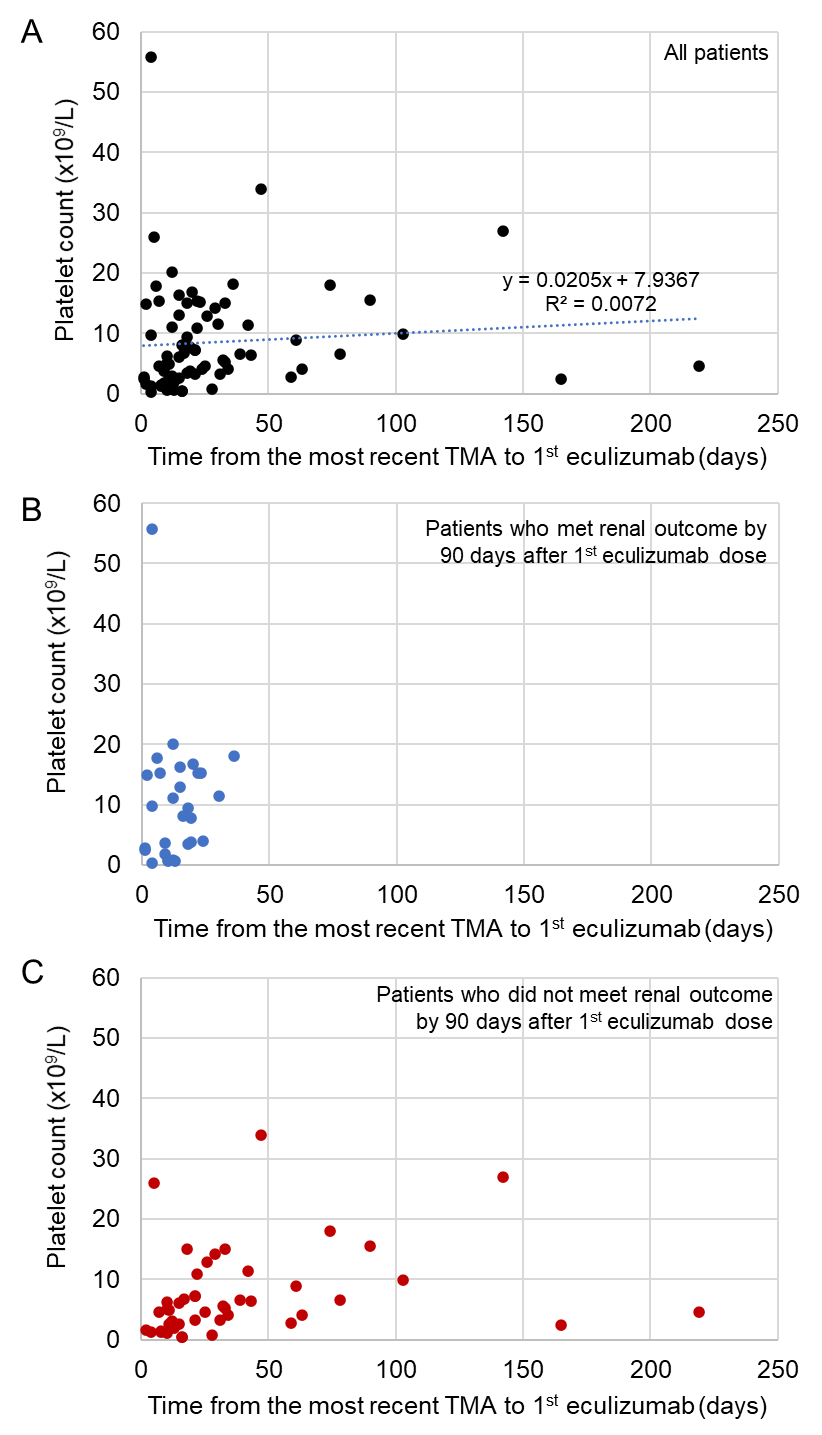
**

**Supplementary Fig. S8.** **Scatter plots of individual patients showing their platelet counts for time from the most recent TMA to 1^st^ eculizumab**

Scatter plots of individual patients of all (A), who met improvement of renal function (>25% sCr from baseline or off-dialysis by 90 days after the initiation of eculizumab) (B) and, who did not meet the renal outcome (C) were generated to show relationship between platelet counts at baseline and time from the most recent TMA to 1^st^ eculizumab. Approximate curve was generated to identify R^2^ in A. TMA, thrombotic microangiopathy.


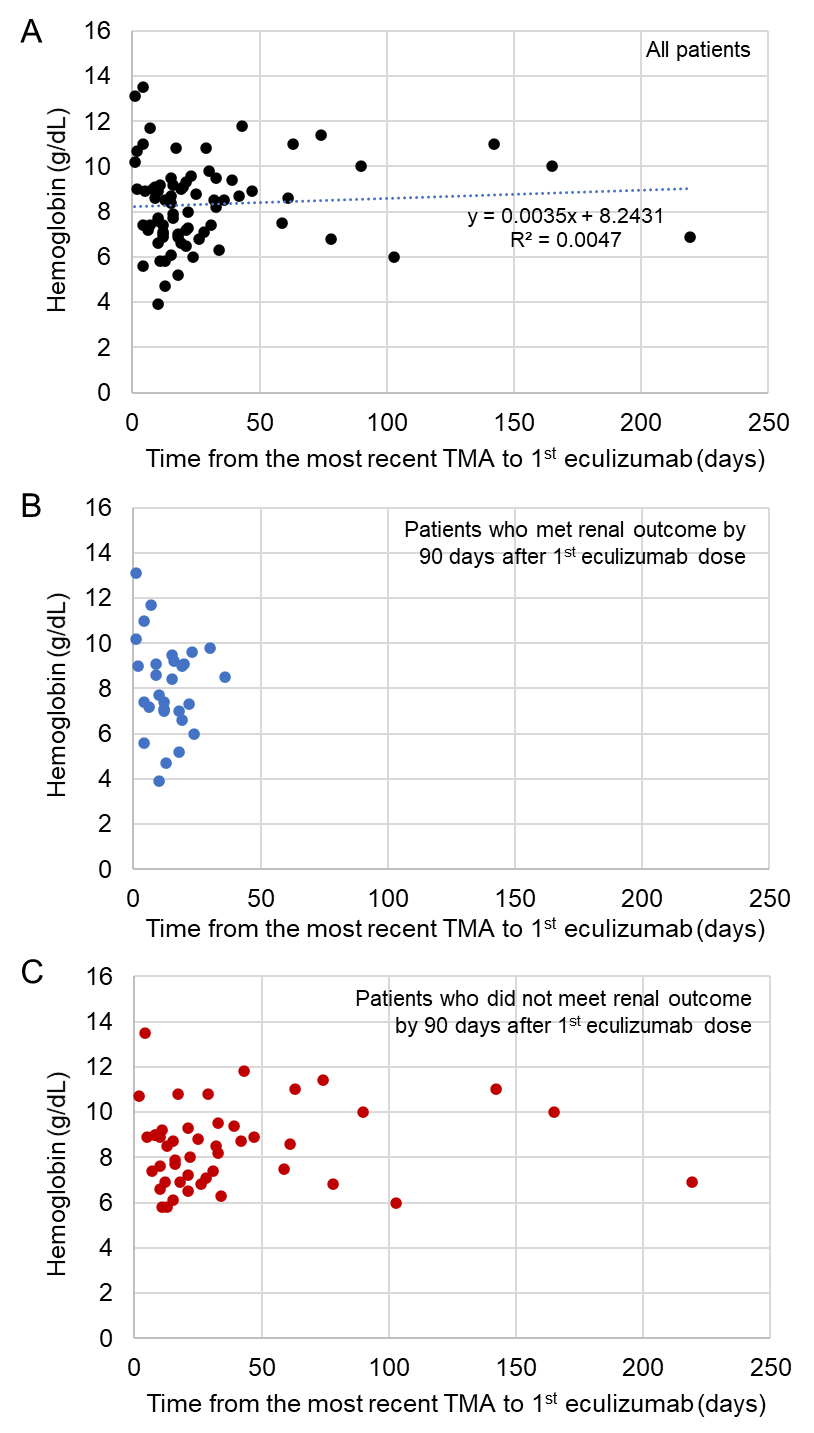


**Supplementary Fig. S9.** **Scatter plots of individual patients showing their haemoglobin values for time from the most recent TMA to 1^st^ eculizumab**

Scatter plots of individual patients of all (A), who met improvement of renal function (>25% sCr from baseline or off-dialysis by 90 days after the initiation of eculizumab) (B) and, who did not meet the renal outcome (C) were generated to show relationship between haemoglobin values at baseline and time from the most recent TMA to 1^st^ eculizumab. Approximate curve was generated to identify R^2^ in A. TMA, thrombotic microangiopathy.

**
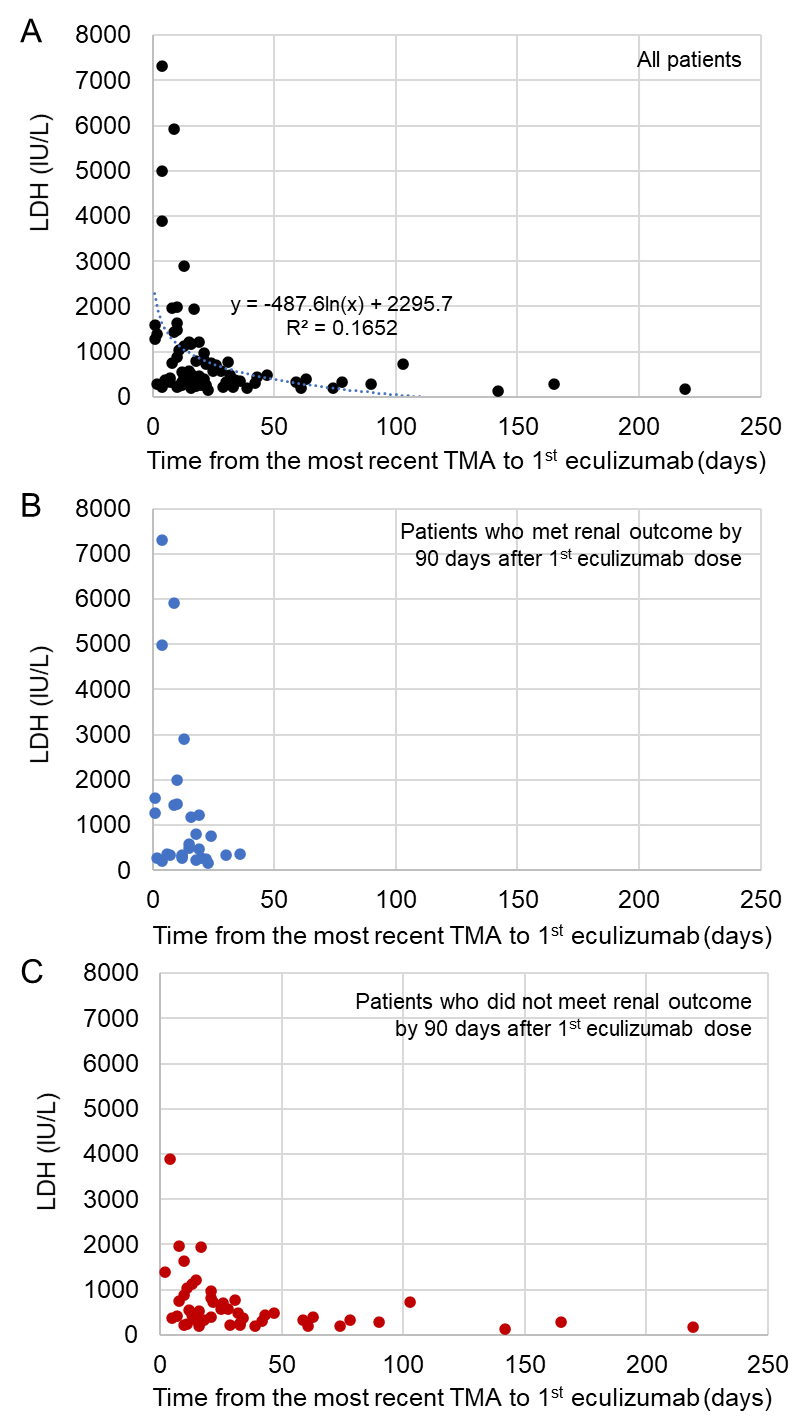
**

**Supplementary Fig. S10.** **Scatter plots of individual patients showing their LDH levels for time from the most recent TMA to 1st eculizumab**

Scatter plots of individual patients of all (A), who met improvement of renal function (>25% sCr from baseline or off-dialysis by 90 days after the initiation of eculizumab) (B) and, who did not meet the renal outcome (C) were generated to show relationship between LDH levels at baseline and time from the most recent TMA to 1^st^ eculizumab. Approximate curve was generated to identify R^2^ in A. LDH, lactate dehydrogenase; TMA, thrombotic microangiopathy.

**
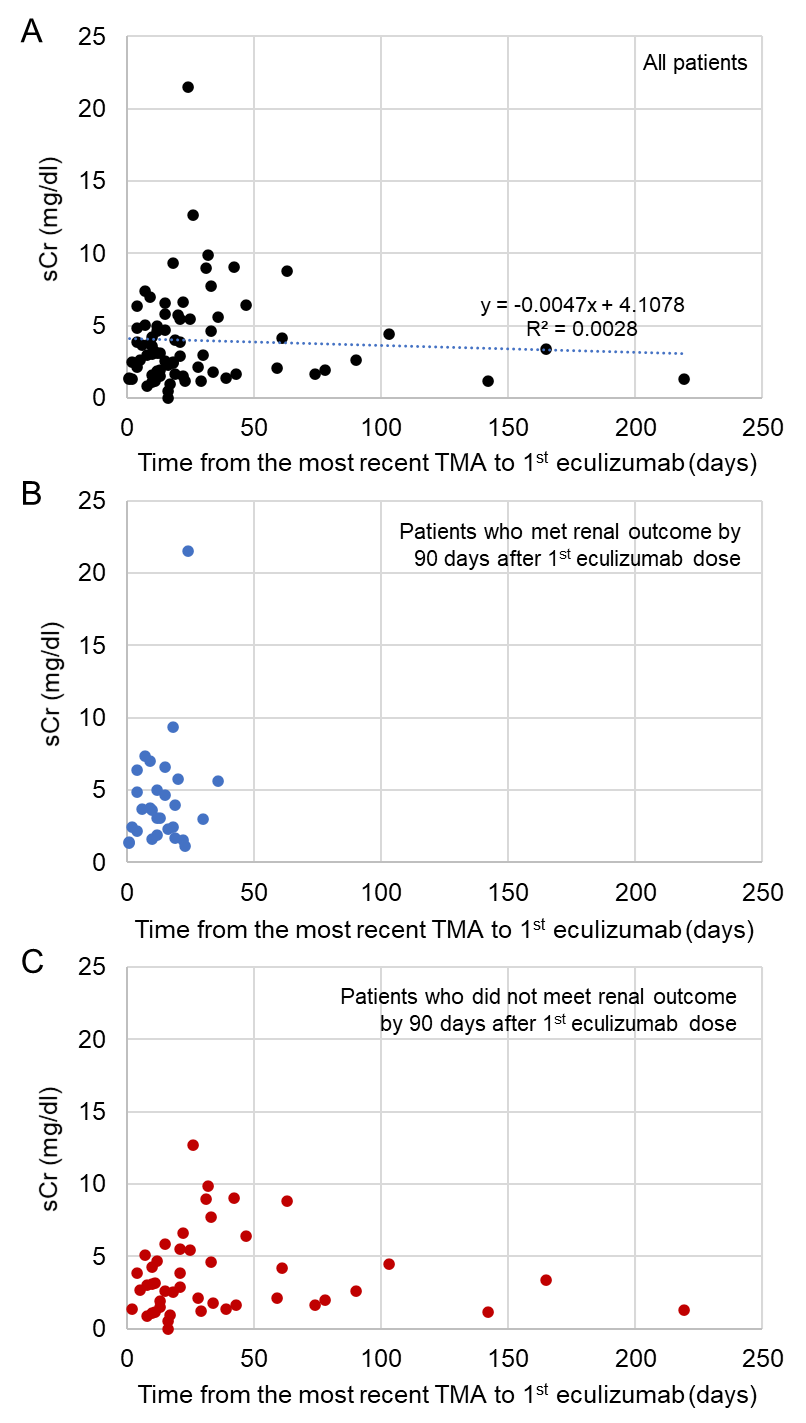
**

**Supplementary Fig. S11.** **Scatter plots of individual patients showing their sCr values for time from the most recent TMA to 1^st^ eculizumab**

Scatter plots of individual patients of all (A), who met improvement of renal function (>25% sCr from baseline or off-dialysis by 90 days after the initiation of eculizumab) (B), and who did not meet the renal outcome (C) were generated to show relationship between sCr values at baseline and time from the most recent TMA to 1^st^ eculizumab. Approximate curve was generated to identify R^2^ in A. sCr, serum creatinine; TMA, thrombotic microangiopathy.

**
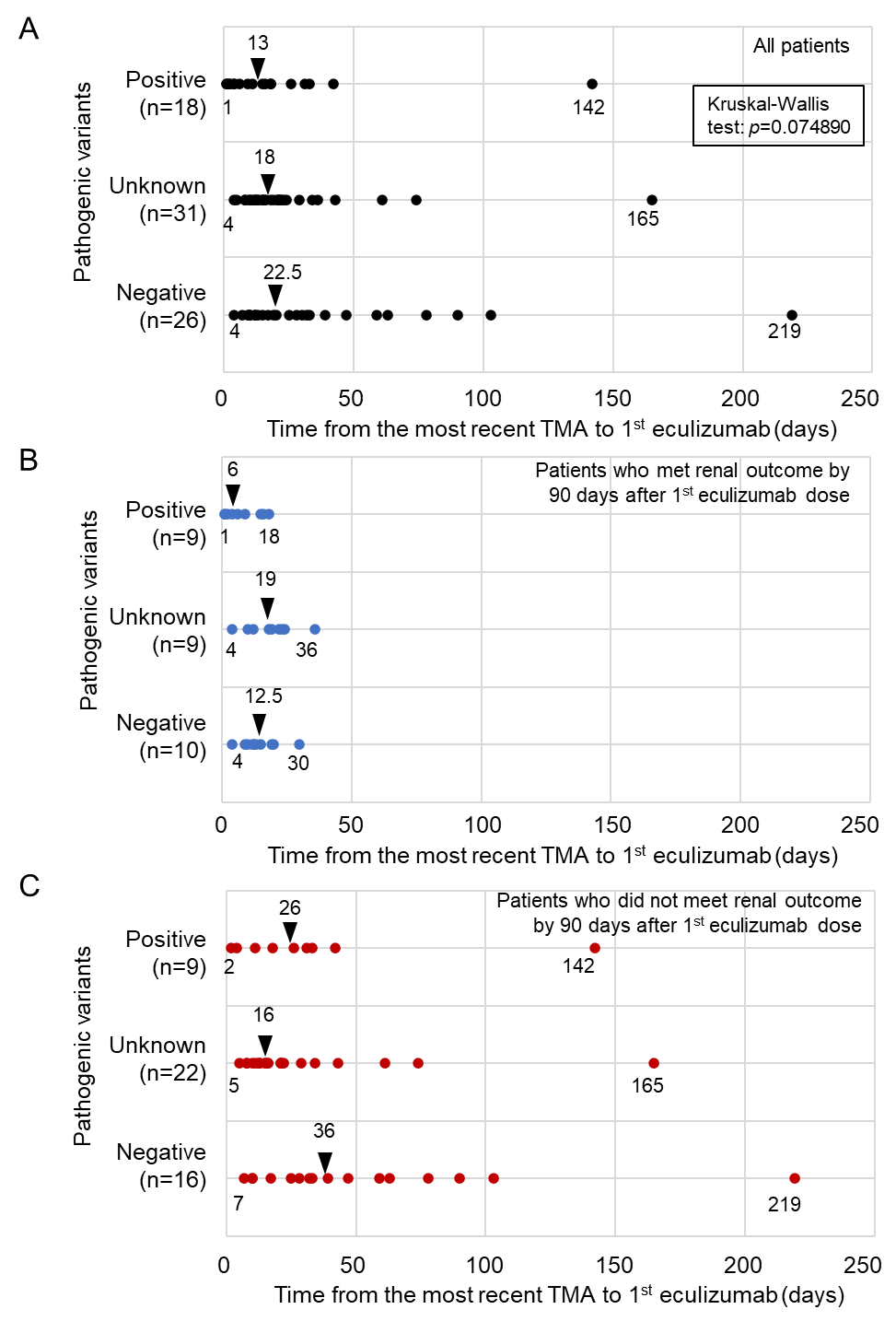
**

**Supplementary Fig. S12.** **Scatter plots of individual patients showing the presence of pathogenic gene variants for time from the most recent TMA to 1^st^ eculizumab**

Scatter plots of individual patients of all (A), who met improvement of renal function (>25% sCr from baseline or off-dialysis by 90 days after the initiation of eculizumab) (B) and, who did not meet the renal outcome (C) were generated to show relationship between the presence of pathogenic gene variants and time from the most recent TMA to 1st eculizumab. Mean time from the most recent TMA to 1^st^ eculizumab was indicated by allow head. The numbers under pots were minimum and maximum days from the most recent TMA to 1st eculizumab. Statistical difference in median time from the most recent TMA to 1st eculizumab between patient groups unknown of, with and without pathogenic gene variant was examined by Kruskal-Wallis test. TMA, thrombotic microangiopathy.
